# Ultrafast Synthesis of Graphene‐Embedded Cyclodextrin‐Metal‐Organic Framework for Supramolecular Selective Absorbency and Supercapacitor Performance

**DOI:** 10.1002/advs.202304062

**Published:** 2023-08-27

**Authors:** Wang Zhang, Zhiqiang Zheng, Liwei Lin, Xi Zhang, Minjun Bae, Jeongyeon Lee, Ju Xie, Guowang Diao, Hyung‐Jun Im, Yuanzhe Piao, Huan Pang

**Affiliations:** ^1^ School of Chemistry and Chemical Engineering Yangzhou University Yangzhou Jiangsu 225002 China; ^2^ Department of Applied Bioengineering Graduate School of Convergence Science and Technology Seoul National University Seoul 08826 South Korea; ^3^ School of Petrochemical Engineering Changzhou University Changzhou Jiangsu 213164 China; ^4^ College of Design Hanyang University Ansan‐si Gyeonggi‐do 15588 South Korea; ^5^ Institute of Textiles and Clothing The Hong Kong Polytechnic University Hung Hom Hong Kong SAR 999077 China

**Keywords:** cyclodextrin, metal–organic framework, microwave, supercapacitor, supramolecular adsorbent

## Abstract

Limited by preparation time and ligand solubility, synthetic protocols for cyclodextrin‐based metal‐organic framework (CD‐MOF), as well as subsequent derived materials with improved stability and properties, still remains a challenge. Herein, an ultrafast, environmentally friendly, and cost‐effective microwave method is proposed, which is induced by graphene oxide (GO) to design CD‐MOF/GOs. This applicable technique can control the crystal size of CD‐MOFs from macro‐ to nanocrystals. CD‐MOF/GOs are investigated as a new type of supramolecular adsorbent. It can selectively adsorb the dye molecule methylene green (MG) owing to the synergistic effect between the hydrophobic nanocavity of CDs, and the abundant O‐containing functional groups of GO in the composites. Following high temperature calcination, the resulting N, S co‐doped porous carbons derived from CD‐MOF/GOs exhibit a high capacitance of 501 F g^−1^ at 0.5 A g^−1^, as well as stable cycling stability with 90.1% capacity retention after 5000 cycles. The porous carbon exhibits good electrochemical performance due to its porous surface containing numerous electrochemically active sites after dye adsorption and carbonization. The design strategy by supramolecular incorporating a variety of active molecules into CD‐MOFs optimizes the properties of their derived materials, furthering development toward the fabrication of zeitgeisty and high‐performance energy storage devices.

## Graphical abstract

A fast microwave method based on a graphene template is proposed to synthesize CD‐MOF/GOs, which are supramolecular adsorbents with good selectivity for methylene green. The design strategy of supramolecular incorporating a variety of active molecules into CD‐MOFs can optimize the properties of their derived carbon materials, making them promising candidates for energy storage devices.

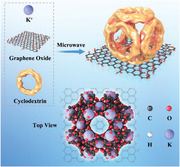

## 1 Introduction

With the rapid development of mobile devices, new energy tools, and vehicles, energy storage has gained significant attention.^[^ ^1^ ^]^ Among various energy storage devices, supercapacitors have emerged as a widely studied option, alongside lithium‐ion batteries. As an advanced type of rechargeable device, supercapacitors combine the advantages of traditional capacitors and batteries, offering high power density and long cycle life.^[^ ^2^ ^]^ The research on supercapacitors has greatly promoted the development of new intelligent devices.^[^ ^3^ ^]^ Namely, double‐layer capacitors, known for their fast and stable energy storage performance as well as environmentally friendly properties, have been widely studied by researchers.^[^ ^4^ ^]^ Additionally, metal‐organic frameworks (MOFs) are one of the most popular electrode materials because of their large pores, flexible preparation methods, and controllable structures.^[^ ^5^ ^]^ The synergistic effect between MOFs and other active factors can further improve the electrochemical properties of supercapacitors.^[^ ^6^ ^]^

The γ‐cyclodextrin (γ‐CD) is a C8‐symmetrical cyclic oligosaccharide composed of eight asymmetric α−1,4‐linked D‐glucopyranosyl residues that possesses a bucket‐shaped cavity, and cyclodextrin‐based metal‐organic frameworks (CD‐MOFs), derived from γ‐CD and potassium ions (K^+^), exhibit several advantages, including the porous structure, renewable property, edibility, and efficient large‐scale production.^[^ ^7^ ^]^ CD‐MOFs have recently emerged as a novel and versatile class of materials that exhibit exceptional properties. They possess porous frameworks embedded within extended structures, showcasing remarkable crystallinity, permanent porosity, and outstanding biocompatibility. The CD‐MOFs can be obtained by diffusing methanol into an aqueous solution of γ‐CD containing KOH.^[^ ^8^ ^]^ Various approaches have been used to synthesize CD‐MOFs, including vapor diffusion,^[^ ^9^ ^]^ hydrothermal methods,^[^ ^10^ ^]^ and microwave‐assisted synthesis.^[^ ^11^ ^]^ However, the reported synthetic strategies negatively suffer from the inefficiency and time‐consuming procedures, posing limitations on their practical applications.^[^ ^12^ ^]^ For example, Hu et al. synthesized CD‐MOFs using a vapor diffusion method over 3–5 weeks;^[^ ^13^ ^]^ whereas Li et al. synthesized CD‐MOFs using a hydrothermal method in 6 h.^[^ ^10^ ^]^ Additionally, Liu et al. used a microwave method to synthesize γ‐CD‐MOFs in 1 h.^[^ ^11^ ^]^ These problems must be addressed during the actual design and application of CD‐MOFs. Combined with the special cavity structure of CDs, CD‐MOFs endow the flexibility in synthesis and design of MOFs, and have been found widespread applications in various fields,^[^ ^14^ ^]^ including template‐based synthesis of nanoparticle,^[^ ^15^ ^]^ electrochemistry,^[^ ^16^ ^]^ drug delivery,^[^ ^17^ ^]^ catalyst supports,^[^ ^18^ ^]^ adsorption, and separation.^[^ ^19^ ^]^

Actually, the CDs with abundant pores and restricted structures are particularly effective in the adsorption of organic dyes by host‐guest interactions.^[^ ^20^ ^]^ While previous studies have shown successful adsorption of single dyes, selectivity for multiple dyes in mixed solutions and a comprehensive understanding of the adsorption mechanism are still lacking.^[^ ^21^ ^]^ However, γ‐CD‐MOFs, due to their large number of O‐containing functional groups and nano‐structured cavities, hold promising prospects in the field of selective adsorption. Notably, the dye adsorption process also introduces N and S doping into the resulting porous carbon materials. The derived porous carbons from CD‐MOFs can be used as precursors for supercapacitor electrodes after high‐temperature calcination. Especially, the derived carbons exhibit a hierarchical porous structure, high specific capacitance, and excellent electrochemical performance.^[^ ^22^ ^]^ In essence, CD‐MOFs serve as ideal precursors for porous carbons due to their tunable frameworks, abundant ligand choices, and superior chemical stability.^[^ ^23^ ^]^

Herein, the microwave method in the synthesis of the γ‐CD‐MOF/graphene oxide (GO) composite has successfully addressed the problem of long preparation time associated with traditional methods. The presence of GO provides numerous nucleation sites, facilitating the formation of γ‐CD‐MOF within a few minutes. The selective adsorption ability of γ‐CD‐MOF/GO toward methylene green (MG) was demonstrated through adsorption experiments using methyl orange (MO), methylene blue (MB), and MG mixed solutions. To optimize the electrochemical performance of the derived carbons, different precursors of γ‐CD‐MOF/GO with varying GO concentrations and calcination temperatures were compared. The final obtained carbon materials exhibited porous structures with a large surface area, which would be conducive to the transport of ions and electrons. After the adsorption process, the specific capacitance of the derived porous carbons increased from 326 to 501 F g^−1^. The adsorption of dyes greatly enhanced the specific capacitance of the derivatives, as well as improved the rate performance and electrochemical kinetics of the supercapacitor.

## 2 Results and Discussion

### 2.1 Structure and Physicochemical Features of γ‐CD‐MOF/GO

**Figure 1:**
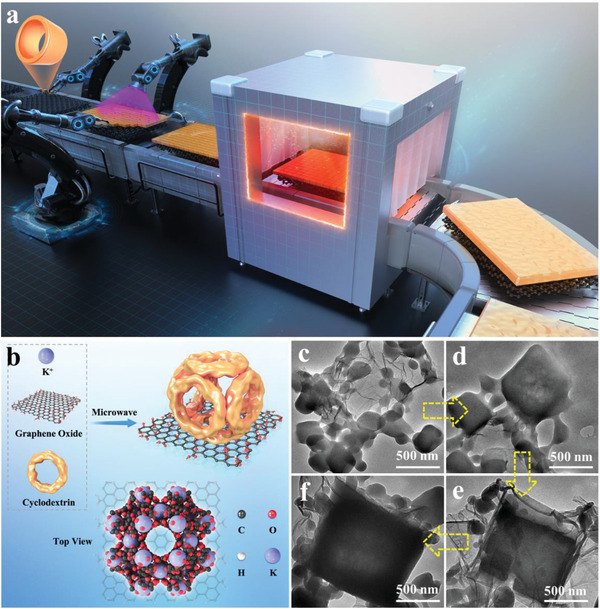
a) Schematic illustrating the fabrication process. b) Formation of γ‐CD‐MOF on the Surface of GO by Microwave. SEM images of γ‐CD‐MOF/GO prepared at different times: c) 0.5, d) 1, e) 3, and f) 4.5 min.

Integrated MOFs with GOs have aroused huge interest in recent years due to their unique properties and good performances compared to either alone.^[^ ^24^ ^]^ From the available literature, this study represents the first report on the synthesis of CD‐MOFs using GO as a heterogeneous nucleating agent and size modulator. Ascribed to the distinctive structural and functional properties of GO, the nucleation and crystallization processes of CD‐MOFs can be promoted in this microwave irradiation method (**Figure** **1**a,b), tailoring γ‐CD‐MOF/GO composites with different nanostructures. Transmission electron microscopy (TEM) images of γ‐CD‐MOF/GO ultrafast synthesized at different time intervals reveal uniform cubic morphologies, as shown in Figure 1c–f. The size of γ‐CD‐MOF crystals (≈1 µm) was smaller when compared with those obtained using CTAB via the vapor diffusion method.^[^ ^25^ ^]^ Further studies were conducted to investigate the effect of reaction time and other parameters on the crystal sizes and morphologies of γ‐CD‐MOFs synthesized using the microwave irradiation method.

One of the critical aspects in the development of MOF/GO composites is to understand the influence of GO on the nucleation and growth of MOF structures. This fundamental research provided vast opportunities to tailor the nanostructures of MOF/GO composites with desirable properties and features, thus targeting many challenging application potentials. Our previous research focused on the nucleation and growth of MOFs, which were significantly influenced by the presence of oxygen‐containing functional groups on the basal planes of GO, as well as the coordination of metallic sites in the parental MOF structures.^[^ ^26^ ^]^ Moreover, GO could participate in the crystallization process of MOFs and control over the direction of MOF growth, leading to oriented MOF structures with uniform crystal and pore structures. And the microwave‐assisted method offered a rapid and efficient approach to produce CD‐MOF on GO, yielding nano‐structured CD‐MOF with a significantly enhanced surface area. The oxygen‐containing groups on GO played a crucial role in promoting CD‐MOF formation, as these groups served as strong binding sites for metal ions and CDs, anchoring them firmly to the graphene surface. Moreover, the size of CD‐MOF was controlled and tailored by adjusting the concentration of GO during the synthesis process. Overall, this method presented a valuable strategy to fabricate nano‐structured CD‐MOF composites.

In this microwave procedure, γ‐CD exhibits an irregular structure without adding GOs or other nucleating agents, because local thermal effects by microwave can also induce the deterioration in cubic structure of CD‐MOFs, as shown in Figures S1a and S2 (Supporting Information). However, when GO is added as a nucleating agent with varying concentrations from 0.1 to 4 g L^−1^, cubic γ‐CD‐MOF/GO crystals are obtained, as shown in Figure S1b–g (Supporting Information). The number of γ‐CD‐MOF crystals increased with higher concentrations of GO, as GO facilitated the formation of numerous nucleation sites. Further, the size of γ‐CD‐MOF illustrated the similar tendency, and the cubic crystals became more regular (Figure S1c–g, Supporting Information). Notably, these features became much more pronounced for γ‐CD‐MOF/GO prepared at GO concentrations of 3 g L^−1^. This phenomenon was ascribed to the coordination between the oxygen groups in GO and K^+^ in the CD‐MOF, which promoted the crystal growth of γ‐CD‐MOF/GOs. Obviously, the synergistic effect of microwave irradiation and GO facilitated the rapid nucleation and growth of MOFs. As shown in Figure S1h (Supporting Information), the crystal size of γ‐CD‐MOF/GO remains relatively unchanged even after the adsorption of dye molecules, maintaining a crystal size of 1–2 µm. Furthermore, the mapping of γ‐CD‐MOF/GO at GO concentrations of 3 g L^−1^ revealed that O, C, and K elements were evenly dispersed (Figure S3, Supporting Information).

The X‐ray diffraction (XRD) results in Figure S5 (Supporting Information) indicate the high crystallinity of γ‐CD‐MOF/GOs prepared at various GO concentrations, which is consistent with the crystals synthesized via the conventional methods.^[^ ^27^ ^]^ The XRD patterns of γ‐CD‐MOF/GO exhibited strong peaks at 2*θ* = 5.7° and 10°, corresponding to the (200) and (400) Bragg diffractions planes of the CD‐MOF crystal, respectively. However, a significant loss in crystallinity was observed at a GO concentration of 4 g L^−1^ despite their cubic shapes. It was worth noting that microwave irradiation induced local heating effects. When GO concentrations exceed the optimum levels, the cubic structure of CD‐MOFs deteriorated due to excessive localized heating caused by the thermal effect of microwaves.^[^ ^11^ ^]^ The morphologies of α‐CD‐MOF/GO and β‐CD‐MOF/GO are characterized in Figure S6 (Supporting Information). Based on the above analysis, it is intriguing to note that in the synthesis of CD‐MOF using the microwave method, the utilization of GO as a nucleating agent demonstrated an important influence. Specifically, as the content of GO increased, it appeared to exert control over the size of the synthesized crystals, transitioning them from macro‐ to nanoscale dimensions.

### 2.2 Adsorption of γ‐CD‐MOF/GO to Dyes

**Figure 2:**
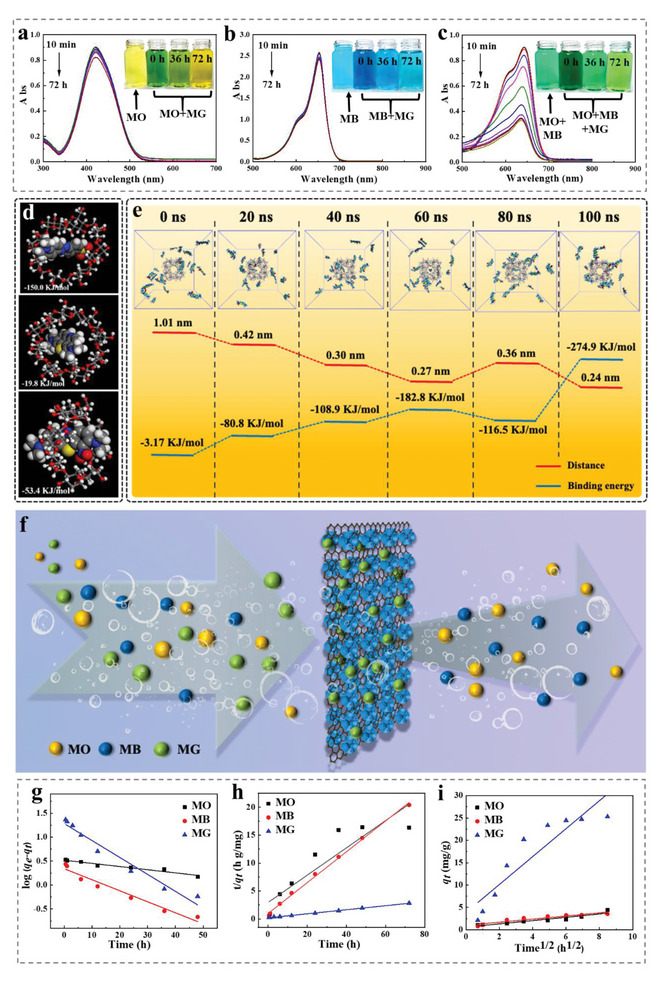
UV–vis spectra of a) 10 mg L^−1^ MO (insets: digital photos of 10 mg L^−1^ MO and MO+MG adsorbed in 0, 36, and 72 h), b) 10 mg L^−1^ MB (insets: digital photos of 10 mg L^−1^ MB and MB+MG adsorbed in 0, 36, and 72 h), c) 10 mg L^−1^ MG (insets: digital photos of 10 mg L^−1^ MO+MB and MO+MB+MG adsorbed in 0, 36, and 72 h. d) Binding energy of γ‐CD to the dye molecules MO, MB, MG. e) Distance and binding energy between the γ‐CD‐MOF and MG within 100 ns. f) Schematic diagram of selective adsorption. g) Pseudo‐first‐order, h) pseudo‐second‐order, i) intraparticle diffusion kinetics model linear relationship for adsorption.

The importance of organic dye‐specific removal behavior in tailoring MOF materials is closely related to the properties of MOFs and the molecular structures of dyes.^[^ ^28^ ^]^ In this study, three common organic dyes with different ionic forms were considered: methylene blue (MB) and methylene green (MG) as cationic dyes with similar chemical structures, and methyl orange (MO) as an anionic dye. The adsorption results for MO, MB, and MG after 72 h are shown in **Figure** **2**a–c, respectively. It was observed that γ‐CD‐MOF/GO exhibited good adsorption capabilities toward MG, as evidenced by the fading of MG color in the solution, while the absorbance of MO and MB solutions only existed minimal changes. Furthermore, when γ‐CD‐MOF/GO was added into mixed solutions containing different combinations of dyes (MO+MG, MB+MG, MO+MB+MG), the color of MG in the solution gradually faded and returned to their original states over 72 h, as shown in the inserted photographs of Figure 2a–c. The UV–vis absorbance changes of the mixed solution of MO and MG within 72 h by γ‐CD‐MOF/GO are shown in Figure S7 (Supporting Information). The absorbance of MG at the wavelength of 640 nm decreased significantly by ≈97%, whereas the characteristic peak intensity of MO at 420 nm remained relatively unchanged. This difference indicated that γ‐CD‐MOF/GO had a much higher adsorption capability toward MG compared with that of MO, indicating that γ‐CD‐MOF/GO exhibited a highly selective adsorption effect toward MG. Similar results were obtained for all combinations of CD‐MOFs and dyes, indicating that γ‐CD‐MOF/GO possessed specific adsorption selectivity toward MG.

The selective adsorption of dyes by γ‐CD‐MOF/GO was primarily controlled by the supramolecular interaction of CDs. The hydrophobic cavity of CDs could form inclusion complexes with guest molecules of specific molecular structures through supramolecular interactions, thereby enhancing the selective adsorption ability. In Figure 2d, the free energy of molecular binding between γ‐CD/MO, γ‐CD/MB, and γ‐CD/MG is calculated as −150.0, −19.8, and −53.4 KJ mol^−1^, respectively. It was expected that MO would be more easily adsorbed by γ‐CD than MB and MG. However, the results found in the γ‐CD‐MOF/GO adsorption environment were opposite, because GO containing rich oxygen groups could inhibit the entry of MO into the cavity of CD.^[^ ^29^ ^]^ This repulsive effect was attributed to the negative charges carried by both MO and GO, as MO was an anionic dye. Although the binding energy of CD to MO was relatively large, the presence of oxygen‐containing functional groups on the surface of GO, such as hydroxyls and esters, lead to suboptimal adsorption effects for MO. On the other hand, MB and MG were both cationic dyes, which were favorable for adsorption by γ‐CD‐MOF/GO.^[^ ^30^ ^]^ Both of the two dyes contain phenothiazine rings, and the greater number of phenothiazine substituents in MG made it larger in size. The cavities of CDs were size‐selective for dyes, with γ‐CD having stronger binding ability to large‐sized dye molecules.^[^ ^31^ ^]^

Combining the digital photos and UV test results in Figure 2a–c, Figures S7 and S8 (Supporting Information), it is concluded that γ‐CD‐MOF/GO has efficient selective adsorption to MG. In order to investigate the adsorption mechanism of γ‐CD‐MOF/GO and MG molecules, the molecular simulation of adsorption was studied. It was found that the interaction between CD and MG molecules was mainly through Van der Waals energy. Further, Figure 2e illustrates γ‐CD‐MOF surrounded by MG molecules within 100 ns. Intermolecular binding energy keeps an increasing trend over time, and the MG molecules gradually gathered from the originally dispersed state to γ‐CD‐MOF. The charge and size of organic dyes were also important factors affecting the selective adsorption.^[^ ^32^ ^]^ Notably, the supramolecular interactions could enhance the adsorption capacity.^[^ ^33^ ^]^ In addition, γ‐CD‐MOF had a large specific surface area and pore volume, endowing γ‐CD‐MOF/GO with an efficient adsorbent for organic or inorganic pollutants. The γ‐CD‐MOF/GO provided a large number of active sites for the dye molecules to enter the material interior, and allowed MG molecules to form a strong bond with the γ‐CD (Figure 2f).

The absorbance of MO, MB, and MG solution is illustrated in Figures S11–13 (Supporting Information). Both the concentrations and absorbance were linearly fitted using Lambert–Beer law. Combining the results, the adsorption capacity of γ‐CD‐MOF/GO to MO, MB, and MG was 4.40 mg g^−1^, 3.53 mg g^−1^, and 25.28 mg g^−1^, respectively, indicating that the adsorption of γ‐CD‐MOF/GO to MG was the best. To investigate the sorption mechanism, three kinds of adsorption kinetic models are studied to reveal the adsorption process (pseudo‐1st‐order, pseudo‐2nd‐order, and intraparticle diffusion), as shown in Figure 2g–i. Table S1 (Supporting Information) illustrates the comparison of the pseudo‐1st‐order, pseudo‐2nd‐order, and intraparticle diffusion adsorption rate constants, and calculated/experimental *q*_e_ values obtained at 25 °C. According to the correlation coefficients, R^2^ for the 2nd‐order kinetic model was higher than those of the 1st‐order kinetic and intraparticle diffusion models, indicating that the adsorption of MG by γ‐CD‐MOF/GO fitted a pseudo‐2nd‐order reaction through a chemisorption process. The adsorption to MB and MG was a combination of weak physical and strong chemical adsorption. Apparently, the adsorption isotherms of MG onto γ‐CD‐MOF/GO at 25 °C are shown in Figure S14 (Supporting Information), as well as the similar results displaying the amount of MG adsorbed. The necessary parameters were fit by the Langmuir and Freundlich isotherm models (Figure S15 and Table S2, Supporting Information). The fitting results manifested that the calculated *q*_max_ value of 28.32 mg g^−1^ was close to the experimental value of 25.28 mg g^−1^, proving that the Langmuir model could better describe the adsorption process. The thermodynamic parameter for the adsorption reaction was determined through the Van't Hoff method.^[^ ^34^ ^]^ A good linear relationship between ln *K*_a_ and 1/*T* was obtained (Figure S16, Supporting Information), and the corresponding values of the molar enthalpy (∆*H* ^0^), entropy change (∆*S* ^0^), and the Gibbs free enthalpy change (∆*G* ^0^) are listed in Table S3 (Supporting Information). With the increase in temperature, more negative values of ∆*G* ^0^ were observed, indicating that higher temperatures favored higher sorption results. The binding affinity of MG molecules toward the CD‐MOF surface also increased with rising temperatures. Further, the positive value of ∆*H* ^0^ revealed that the adsorption process was endothermic in nature, and could be classified into chemisorption since all ∆*H* ^0^ values fell into the range of 20.9–418.4 kJ mol^−1^. Comparatively, the positive value of ∆*S* ^0^ denoted the increased randomness at solid–solution interface during dye adsorption.^[^ ^35^ ^]^

The wide range of suitable CD precursors and organic dyes offered numerous combinations for utilizing CD‐MOF in potential waste liquid treatment. The utilization of GO templates and microwave irradiation significantly influenced the size and shape of the final CD‐MOFs, making it a powerful tool to tailor adsorption characteristics. Another essential advantage of CD‐MOF/GO over other adsorbents was the accessibility to advanced supercapacitor design. However, the diverse reactivity of different dyes toward a specific adsorbent complicated post‐processing and recycling, particularly in the case of CD‐MOF. On the other hand, the GO template executed in CD‐MOF synthesis was comparatively hopeful to convert adsorbed waste (CD‐MOF/GO and dyes) into a high‐performance supercapacitor with CD‐MOF. A proposed method involved using a carbonization activation process to convert γ‐CD‐MOF/GO/MG into 3D interconnected porous carbon. It was noteworthy that using different dyes presents an elegant approach for preparing functional supercapacitor materials with high intrinsic electrical conductivity, large specific surface areas, and abundant heteroatom doping sites.

### 2.3 Structure and Physicochemical Features of γ‐CD‐MOF/GO‐n

**Figure 3:**
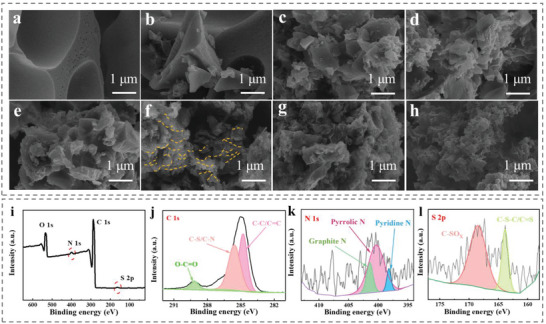
SEM images of porous carbon γ‐CD‐MOF/GO‐600 prepared at different GO concentration: a) 0, b) 0.1, c) 0.5, d) 1, e) 2, f) 3, g) 4 g L^−1^; h) γ‐CD‐MOF/GO/MG‐600. i) XPS spectrum of γ‐CD‐MOF/GO/MG‐600, j) C 1s peaks, k) N 1s peaks, and l) S 2p peaks.

Advanced supercapacitor electrodes required the development of functional materials with conductive and porous skeletons embedded by dense redox sites. Figure S17 (Supporting Information) illustrates the effective sites of γ‐CD‐MOF after the adsorption to MG. The heat treatment of CD‐MOF/GO under an N_2_ atmosphere resulted in the formation of nanostructured porous carbon. The influence of GO concentrations as precursors is also investigated at 600 °C, and the micro‐morphology of the γ‐CD‐MOF/GO‐derived porous carbon is depicted in **Figure** **3**a–h. The initially uniform‐shaped CD‐MOF was broken during carbonization, leading to the formation of a micron‐sized irregular porous structure (Figure 3a). It was noteworthy that CD‐MOF could be fragile without graphene assistance at high temperatures.^[^ ^36^ ^]^ As the GO concentration increased, nanostructured porous carbon gradually generated (Figure 3b–g). The calcined carbon was assembled on a graphene template, forming a 3D porous structure, where the wrinkled graphene was visible and marked by yellow dotted lines (Figure 3f). The GO structure effectively prevented the collapse of the nano porous structure of CD‐MOF during the calcination process, indicating that the graphene template played an important role in supporting the nano‐framework.

In addition, α‐CD and β‐CD were also selected to verify the feasibility of the synthesis of CD‐MOFs and porous carbons with graphene (Figure S18, Supporting Information). Here, it was concluded that the different kinds of CD had no obvious effect on the derived porous carbon structure, and this synthetic method was considered widely applicable. Furthermore, after the adsorption of MG dye and carbonization, γ‐CD‐MOF/GO/MG‐600 also had a 3D network structure (Figure 3h), as well as γ‐CD‐MOF/GO derived porous carbons, indicating that γ‐CD‐MOF/GO following the above adsorption processes did not destroy its structure, maintaining the nanostructure of the derived porous carbon during calcination. From the scanning electron microscopy (SEM) images, it was concluded that the addition of GO could control the size of the porous carbon to the nano size, significantly increasing the specific surface area of porous carbon, which benefited the rapid migration of ions during the electrochemical process.

Additionally, the porous carbons from γ‐CD‐MOF/GO prepared at different temperature (γ‐CD‐MOF/GO‐n) were investigated by Raman spectroscopy to show the broad and overlapping D and G bands at 1350 cm^−1^ and 1580 cm^−1^. These bands existed in the first order Raman spectra in Figure S21 (Supporting Information). Typically, the D band reflected amorphous carbon or defecting structure, while the G bands were attributed to the ideal symmetrical vibrations of the graphitic lattice.^[^ ^37^ ^]^ The intensity ratio of I_D_/I_G_ was used to express the degree of surface defects.^[^ ^38^ ^]^ Further, the strength ratio I_D_/I_G_ of γ‐CD‐MOF/GO‐600 (0.841) was higher than others, indicating that its surface was relatively disordered, and the structure of γ‐CD‐MOF/GO‐600 was obviously damaged in a high temperature, which was both notably consistent with above SEM images (Figure S20, Supporting Information). The presence of obvious surface defects in γ‐CD‐MOF/GO‐600 could promote ion transfer during electrochemical processes and improve electrical conductivity. The Raman spectrum of γ‐CD‐MOF/GO/MG‐600 is shown in Figure S22 (Supporting Information). The *I*_D_/*I*_G_ of γ‐CD‐MOF/GO/MG‐600 was 0.830, lower than the result of γ‐CD‐MOF/GO‐600 (0.841), which illustrated that N, S co‐doping had slight influence on surface defects. However, an appropriate number of heteroatoms could provide abundant electrochemically active sites and enhance the electrochemical performance.^[^ ^39^ ^]^

Adsorption–desorption isotherms of nitrogen at 77 K were used to evaluate the specific surface areas of γ‐CD‐MOF/GO‐n. From γ‐CD‐MOF/GO‐300 to γ‐CD‐MOF/GO‐700 (Figure S23, Supporting Information), the isotherm profiles could be categorized as type IV curves, which possessed mainly micropores and partial meso‐ or macropores based on the IUPAC classification.^[^ ^40^ ^]^ Especially, γ‐CD‐MOF/GO‐600 presents the largest adsorption isotherms section, indicating the existence of more pores of smaller sizes than other samples. The BET specific surface area and average pore size of γ‐CD‐MOF/GO‐n are listed in Table S4 (Supporting Information). The γ‐CD‐MOF/GO‐600 had the largest BET specific surface area of 594.61 m^2^ g^−1^, and its average pore size was relatively small (2.7 nm). The large specific surface area and hierarchical porous structure could greatly improve the electrical conductivity and energy storage capacity.

The composition and valence state of each element in the γ‐CD‐MOF/GO‐n were studied via XPS. Figure S24 (Supporting Information) shows the XPS spectrum of γ‐CD‐MOF/GO prepared with different temperature, where the γ‐CD‐MOF/GO‐derived carbon mainly contained C and O, while the proportion of C increased with temperature. Whereas the proportion of O decreased because during the heating process, O was prone to chemical reactions, hence leading to its loss. C and O contents of γ‐CD‐MOF/GO prepared with different temperature are also listed in Table S4 (Supporting Information). The XPS spectra demonstrate that N and S co‐doping occurred in γ‐CD‐MOF/GO/MG‐600 through adsorption and carbonization processes (Figure 3i). It could be intuitively seen that N and S elements were successfully entered structurally, which was beneficial to improve the electronic structure.^[^ ^35^, ^41^ ^]^ Figure 3j–l illustrate the C 1s, N 1s, and S 2p high‐resolution spectra of γ‐CD‐MOF/GO/MG‐600, respectively. Among them, C had three main peaks of C─C/C = C at 284.8 eV, C─S/C─N at 285.3 eV, and O─C = O at 289 eV. Further, N displays peaks of pyridine N at 398.2 eV, pyrrolic N at 400 eV, and graphitic N at 401.4 eV. Lastly, S mainly showed two peaks: C─S─C/C = S at 163.6 eV, and C─SO_X_ at 168.4 eV. The co‐doping of N and S could enhance the wettability of materials, which also provided more active sites and promoted efficient synergy, thereby improving the electrochemical performance.^[^ ^42^ ^]^

### 2.4 Electrochemical Performance

**Figure 4:**
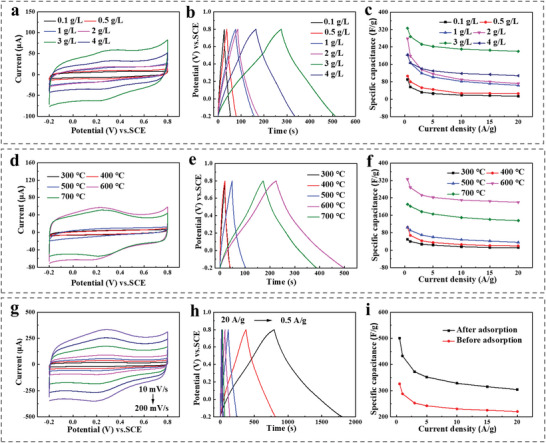
a) CV curves (scan rates, 50 mV s^−1^), b) GCD curves (current densities: 1 A g^−1^), c) Specific capacitance at different current densities (0.5–20 A g^−1^) of porous carbon γ‐CD‐MOF/GO‐600 (derived from γ‐CD‐MOF/GO with different GO concentrations: 0.1, 0.5, 1, 2, 3, and 4 g L^−1^). d) CV curves (scan rates, 50 mV s^−1^), e) GCD curves (current densities: 1 A g^−1^), f) Specific capacitance at different current densities (0.5–20 A g^−1^) of porous carbon γ‐CD‐MOF/GO‐n with different temperature (300, 400, 500, 600, 700 °C) in 1 mol L^−1^ H_2_SO_4_. g) CV curves at different scan rates (10–200 mV s^−1^), h) GCD curves at different current densities (0.5–20 A g^−1^) of the electrode modified by γ‐CD‐MOF/GO/MG‐600, i) Specific capacitance at different current densities (0.5–20 A g^−1^) of the electrodes modified by γ‐CD‐MOF/GO‐600 and γ‐CD‐MOF/GO/MG‐600 in 1 mol L^−1^ H_2_SO_4_.

To confirm the effect of GO, the electrochemical properties of γ‐CD‐MOF/GO‐600 prepared with different concentrations of GO are measured in **Figure** **4**a–c. It was observed that the chemical energy storage performance of γ‐CD‐MOF/GO‐600 improved with increasing GO concentrations, and the sample prepared with 3 g L^−1^ GO exhibited the highest specific capacitance. This result suggested a favorable compatibility between the nanostructure of porous carbons and the size of electrolyte ions, leading to the improvement of electrochemical performance.

To evaluate the influence of calcination temperature on the electrochemical performances of γ‐CD‐MOF/GO prepared at 3 g L^−1^ GO, the CV and GCD curves are analyzed in Figure 4d–f. The results demonstrated that higher calcination temperatures (600 and 700 °C) led to improved electrochemical properties of the porous carbons. The higher capacitance values observed were attributed to the larger BET surface area of γ‐CD‐MOF/GO obtained at higher temperatures compared with those obtained at lower temperatures. Notably, γ‐CD‐MOF/GO‐600 exhibited exceptional behavior, retaining 67.5% of its specific capacitance even at a high current density of 20 A g^−1^. The unique structure combining micro‐ and mesopores structure with a large surface area, along with good electronic conductivity at suitable calcination temperatures, made γ‐CD‐MOF/GO become ideal for advanced electrochemical capacitors.

**Figure 5:**
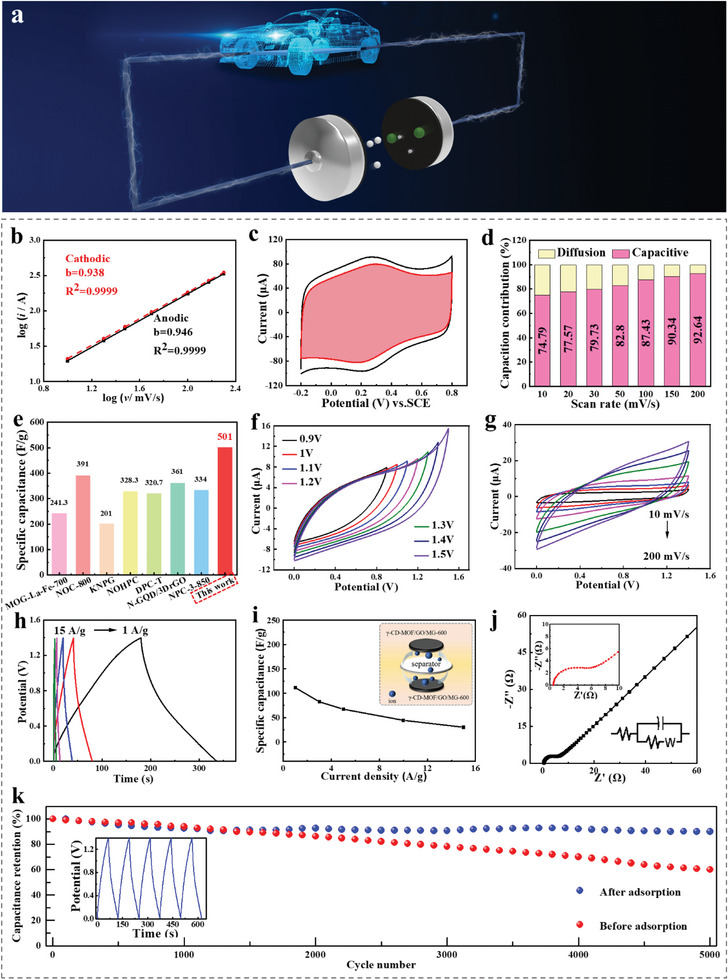
a) Schematic diagram of symmetrical supercapacitor. b) Determination of the anodic and cathodic b‐values, c) CV curves of capacitive and diffusion‐controlled contributions (red shadow) to charge storage at a scan rate of 50 mV s^−1^, d) Capacitive contributions at different scan rates (10–200 mV s^−1^) of γ‐CD‐MOF/GO/MG‐600 in 1 mol L^−1^ H_2_SO_4_. e) Specific capacitance of various reported carbon‐based cathode materials. f) CV curves at different voltages (scan rate, 50 mV s^−1^), g) CV curves at different scan rates (10–200 mV s^−1^), h) GCD curves at different current densities (1–15 A g^−1^), i) Specific capacitance at different current densities (1–15 A g^−1^), j) EIS Nyquist plots (insets: impedance circuit diagram and magnified high‐frequency region of γ‐CD‐MOF/GO/MG‐600//γ‐CD‐MOF/GO/MG‐600), k) Long‐term cycling stability of γ‐CD‐MOF/GO‐600//γ‐CD‐MOF/GO‐600 and γ‐CD‐MOF/GO/MG‐600//γ‐CD‐MOF/GO/MG‐600 over 5000 GCD cycles at a current density of 5 A g^−1^ (inset: GCD curves of γ‐CD‐MOF/GO/MG‐600//γ‐CD‐MOF/GO/MG‐600 recorded in last cycles).

As mentioned above, the adsorption analyses demonstrated the high selectivity of γ‐CD‐MOF toward MG, suggesting that this azo dye could potentially serve as a source of guest molecules for multi‐heteroatom (N and S) doping of CD‐MOF through host‐guest reactions. The resulting porous material (γ‐CD‐MOF/GO/MG‐600) after high temperature calcination was then used as electrode material for supercapacitors. Additionally, Figure 4g illustrates the CV curves of γ‐CD‐MOF/GO/MG‐600 across different scan rates of 10–200 mV s^−1^. Even at a large scan rate (200 mV s^−1^), the CV curve still sustained a quasi‐rectangular shape with weak humps, thus indicating the combination of typical EDLC behaviors and limited redox reactions, which was contributed by the heteroatom incorporation. Further, Figure 4h illustrates the GCD curves of γ‐CD‐MOF/GO/MG‐600 at different current densities, which are similar isosceles triangle shapes, confirming the EDLC behavior. Compared with other samples at the same current densities, γ‐CD‐MOF/GO/MG‐600 presented longer charge and discharge times than other samples at same current densities, illustrating its better capacitive performance. Moreover, the GCD plots at higher current densities maintained a triangular shape, revealing its high reversibility. Thus, the maximum specific capacitance acquired for γ‐CD‐MOF/GO/MG‐600 was 501 F g^−1^; whereas for γ‐CD‐MOF/GO‐600, it was 326 F g^−1^ at 0.5 A g^−1^. Furthermore, the electrochemical performance of γ‐CD‐MOF/GO/MG‐600 was enhanced through N and S‐doping by MG, as evidenced by the superior performance at different current densities and stable retention at high current densities. The specific capacitances of the synthesized materials were compared with those of recently reported composite materials in Figure 4i (specific values listed in Table S6, Supporting Information). For the improvement of electrochemical performance, N doping in γ‐CD‐MOF/GO/MG‐600 created additional defects and active sites within its structure, leading to enhance electronic conductivity and improve surface wettability. These effects were beneficial for boosting the supercapacitor performance. On the other hand, S doping also played a significant role in electrochemical reactions and increased the layer spacing, which facilitated efficient ion storage. As a result, the combination of N and S doping synergistically improved the overall performance of supercapacitors.^[^ ^43^ ^]^ Thus, the results showed that the materials obtained in this research exhibited larger specific capacitances and superior electrochemical performances, making them highly promising for the next generation energy storage products (**Figure** **5**a).^[^ ^44^ ^]^

In order to investigate the charge storage mechanism, Dunn's method was used to examine the CV profiles of γ‐CD‐MOF/GO/MG‐600 from 10 to 200 mV s^−1^. The results exhibited good rate capability and kinetic performance of γ‐CD‐MOF/GO/MG‐600, with fitted slope b values approaching 1.0 in H_2_SO_4_, indicating that capacitive behavior was dominant in the electrochemical process (Figure 5b). The *b* values were calculated for specific potentials based on the CV voltage windows in Figure 5c. Further, the capacitive contributions of γ‐CD‐MOF/GO/MG‐600 appeared a gradually increasing trend from 74.79% to 92.64% as the scan rates increased from 10 to 200 mV s^−1^, indicating the dominant contribution of capacitive storage (Figure 5d).

A symmetric two‐electrode system was fabricated and denoted as γ‐CD‐MOF/GO/MG‐600//γ‐CD‐MOF/GO/MG‐600. Figure 5f shows the CV curves within different working voltages, revealing similar rectangular profiles with no obvious polarization, as well as the emergence of a small current leap when the potential exceeds 1.4 V. Furthermore, CV tests in Figure 5g demonstrated that γ‐CD‐MOF/GO/MG‐600 displayed great stability across different scan rates. The GCD curves were nearly symmetrical, as shown in Figure 5h. At a current density of 1 A g^−1^, the specific capacitance reached 111.3 F g^−1^. Nyquist plots were recorded and fitted in Figure 5j, revealing a low interfacial *R*_ct_ of 2.9 Ω. Energy densities and power densities of γ‐CD‐MOF/GO/MG‐600 have been shown in Figure S28 (Supporting Information). The maximum energy density obtained from this device was 152.8 W h kg^−1^, and the power density was 3500 W kg^−1^.

Additionally, the N, S co‐doping strategy had a significant positive effect on the improvement of material stability, as shown in Figure 5k. After 5000 cycles of charge and discharge, the capacitance stability of γ‐CD‐MOF/GO/MG‐600 reached 90.1%, whose substantially was higher than that of γ‐CD‐MOF/GO (60.03%). The superior electrochemical performance of γ‐CD‐MOF/GO/MG hybrids was attributed to several factors: (1) the GO‐induced CD‐MOF strategy that enhanced charge storage performance; (2) graphene acted as a stable framework with abundant growth sites during CD‐MOF calcination, preventing the collapse of MOF porous structures and providing stable conductive paths in electrochemistry; (3) the supramolecular interactions between the dye molecules and CD‐MOF, which promoted the integration of N and S heteroatom sites with CD‐MOF derived carbons. These synergistic effects of co‐doping heteroatoms held considerable promise for improved electrochemical performances.

## 3 Conclusion

In summary, the present study highlights the ultrafast, low‐cost, and facile method for synthesizing CD‐MOF/GOs. With the support of absorbency investigations and DFT calculations, the remarkable performance of γ‐CD‐MOF/GO was assigned to the selective adsorption for MG occurring from the synergistic effects between hydrophobic nano‐cavity of CDs, and abundant O‐containing functional groups of GOs. As proof‐of‐sustainable production, symmetric supercapacitor with CD‐MOF/GO derived porous carbon was also assembled, delivering a good capacitance of 111.3 F g^−1^ at 1 A g^−1^. This work provided insightful guidelines for exploring selectively adsorption in new CD framework materials, which could benefit the development of dye wastewater treatment and energy storage.

## Conflict of Interest

The authors declare no conflict of interest.

## Supporting information

Supporting Information

## Data Availability

Research data are not shared.
