# Supplementary material for: Ultrafast Synthesis of Graphene‐Embedded Cyclodextrin‐Metal‐Organic Framework for Supramolecular Selective Absorbency and Supercapacitor Performance
Source: Adv Sci (Weinh). 2023 Aug 27;10(31):2304062. doi: 10.1002/advs.202304062 (PMC10625068; doi:10.1002/advs.202304062)
Supplement: Supplementary file 1 — Supporting Information [file ADVS-10-2304062-s001.pdf]

ADVANCED SCIENCE
Open Access

Supporting Information

for *Adv. Sci.*, DOI 10.1002/advs.202304062

Ultrafast Synthesis of Graphene-Embedded Cyclodextrin-Metal-Organic Framework for Supramolecular Selective Absorbency and Supercapacitor Performance

Wang Zhang, Zhiqiang Zheng, Liwei Lin, Xi Zhang, Minjun Bae, Jeongyeon Lee, Ju Xie, Guowang Diao, Hyung-Jun Im, Yuanzhe Piao* and Huan Pang**

Supporting Information

Ultrafast Synthesis of Graphene-Embedded Cyclodextrin-Metal-Organic Framework for Supramolecular Selective Absorbency and Supercapacitor Performance

Wang Zhang, Zhiqiang Zheng, Liwei Lin, Xi Zhang, Minjun Bae, Jeongyeon Lee, Ju Xie, Guowang Diao, Hyung-Jun Im, Yuanzhe Piao*, and Huan Pang**

W. Zhang, Z. Zheng, J. Xie, G. Diao, H. Pang
School of Chemistry and Chemical Engineering
Yangzhou University
Yangzhou, Jiangsu 225002, China
E-mail: huanpangchem@hotmail.com; panghuan@yzu.edu.cn

L. Lin, M. Bae, H. Im, Y. Piao
Department of Applied Bioengineering
Graduate School of Convergence Science and Technology
Seoul National University
Seoul, 08826, Korea
E-mail: lin-official@snu.ac.kr, parkat9@snu.ac.kr

L. Lin
School of Petrochemical Engineering
Changzhou University
Changzhou, Jiangsu 213164, China

X. Zhang
College of Design
Hanyang University
Ansan-si, Gyeonggi-do, 15588, Korea

J. Lee
Institute of Textiles and Clothing
The Hong Kong Polytechnic University
Hung Hom, Hong Kong SAR, 999077, China

1. Supplementary discussion

1.1 Experimental part

1.1.1 Materials and instruments

Alpha-cyclodextrin (α-CD), beta-cyclodextrin (β-CD), gamma-cyclodextrin (γ-CD), KOH, methanol, HCI, H_2SO_4, methyl orange (MO), methylene blue (MB), and methylene green (MG) were analytically pure and purchased from Shanghai Aladdin Reagent Co., Ltd (China). Graphene oxide (GO) was prepared using the Hummers method. Deionized water was used throughout the experiment.

Transmission electron microscopy (TEM) images were obtained using a JEM-2100 microscope with an accelerating voltage of 120 kV. Zeiss_Supra55 emission scanning electron microscopy (SEM) was used for the morphological characterization of materials. The XPS information of materials was obtained by X-ray electron spectrometer (ESCALAB 250Xi). The Raman of materials was measured by a laser confocal Raman spectrometer (INVIA REFLEX). Surface area was measured via nitrogen adsorption–desorption isotherms on a Micromeritics ASAP 2020 under a nitrogen atmosphere, with a heating rate of 10°C·min^{-1} using the Brunner-Emmett-Teller (BET) method. A UV-vis photometer (TU-1901) was used to test the UV information of dye solutions.

1.1.2 Preparation of γ-CD-Metal–Organic Framework (MOF)/GO

A combination of 0.6 mmol of γ-CD and 4.8 mmol KOH was placed in a beaker. Then, GO and H_2O were added to prepare 12 mL of mixed solutions of different GO concentrations (0.1, 0.5, 1, 2, 3, and 4 g·L^{-1}). The mixture of γ-CD, KOH and GO was sonicated for 5 min to disperse uniformly. After stirring, 12 mL methanol was added into mixture of γ-CD, KOH, and GO, and stirred again for 1 h. Subsequently, the microwave method was used to generate crystal growth, and the reaction was carried out under 100 W for 4 min 30 s. After microwave exposure, the reactant was added dropwise into a large amount of methanol solution used as a size regulator while still hot. The grown crystals were centrifuged at 7000 rpm, and washed three times with methanol. They were then dried under vacuum at 50°C for 12 h. After removal, the product γ-CD-MOF/GO was obtained by lightly grinding the crystals.

The α-CD-MOF/GO or β-CD-MOF/GO composite was prepared according to the above method, only replacing γ-CD with α-CD or β-CD.

1.1.3 Adsorption of γ-CD-MOF/GO to dyes

First, 10 mg·L^{-1} MO, MB, and MG were prepared in methanol solutions. Then, 5 mg of γ-CD-MOF/GO was placed in a methanol solution of dye molecules. The absorbance for the solution was tested at room temperature, and adsorption took place for a total of 72 h. The MO,

MB, and MG solutions were monitored during the adsorption process via a UV-vis photometer. The γ-CD-MOF/GO/MG was obtained by the adsorption of γ-CD-MOF/GO to MG for 72 h. The γ-CD-MOF/GO/MG was washed with methanol, and dried under vacuum at 50°C for 12 hours.

1.1.4 Preparation of γ-CD-MOF/GO-n and γ-CD-MOF/GO/MG-600 porous carbons

An appropriate amount of precursors γ-CD-MOF/GO was heated to 300, 400, 500, 600, and 700°C at a heating rate of 1°C·min^{-1} in an atmosphere of Ar gas, and held at that temperature for 2 h, before being allowed to naturally cool to room temperature. The initial product was removed, etched with 1 mol·L^{-1} HCI solution, and washed with water three times. The washed product was vacuum dried at 70°C for 12 h. After drying, the derived porous carbon γ-CD-MOF/GO-n was obtained (n is temperature). Similarly, γ-CD-MOF/GO/MG-600 was obtained by heating γ-CD-MOF/GO/MG at 600°C for 2 h.

1.1.5 Preparation of modified electrodes

All electrochemical tests were performed on a Chenhua CHI 760E instrument. In the three-electrode system, 1 mol·L^{-1} H_2SO_4 solution was used as the electrolyte, while saturated calomel electrode was used as the reference electrode, and platinum wire was used for the counter electrode. The working electrode was a glassy carbon electrode modified with 5×10^{-6} L of carbon material aqueous solution with a concentration of 1 mg·mL^{-1}. For the two-electrode system, carbon material, acetylene black, and polyvinylidene fluoride were mixed into a slurry at a mass ratio of 8:1:1. The prepared slurry was coated on titanium foil as the working electrode of the symmetric supercapacitor. Lastly, 1 mol·L^{-1} H_2SO_4 solution was used as the electrolyte, and a filter paper served as a separator.

1.2 Calculation of specific capacitance and adsorption capacity

The Lambert-Beer law for linear fitting is as following formula:

$$A = \varepsilon \cdot c \cdot d \quad \text{(Equation S1)}$$

Where, A represents absorbance; ε represents molar absorption coefficient (L/mg cm); c represents solution concentration (mg/L); d represents UV light path length (cm).

The adsorption capacity can be calculated by the following formula：

$$q_e = \frac{v(C_0 - Ce)}{m} \quad \text{(Equation S2)}$$

Where, q_e represents the adsorption capacity of an adsorbent to dye when the adsorption equilibrium is reached (mg/g); c_0 represents the initial concentration of the dye solution (mg/L); c_e represents the concentration of the dye solution when the adsorption equilibrium is reached (mg/L); *V* represents the solution volume (mL); m represents the adsorbate mass (mg).

The quasi-first-order adsorption kinetic model is as follows:

$$log(q_e - q_t) = log\, q_e - \frac{k_f}{2.303} t \quad \text{(Equation S3)}$$

Where, q_e represents the equilibrium adsorption capacity (mg/g); q_t represents the adsorption capacity when the time is t (mg/g); k_f is the first-order adsorption rate constant (/h); t represents time (h).

The quasi-second-order adsorption kinetic model is as follows:

$$\frac{t}{q_t} = \frac{1}{k_s q_e^2} + \frac{t}{q_e} \quad \text{(Equation S4)}$$

Where, q_e represents the equilibrium adsorption capacity (mg/g); q_t represents the adsorption capacity when the time is t (mg/g); k_s is the second-order adsorption rate constant (g/mg/h); t represents time (h).

The intraparticle diffusion model is as follows:

$$q_t = k_w t^{\frac{1}{2}} + C \quad \text{(Equation S5)}$$

Where, q_t represents the adsorption capacity when the time is t (mg/g); k_w is the intraparticle diffusion model rate constant (mg/g/$h^{1/2}$); t represents time (h); C is a constant with respect to thickness, boundary layer.

The Freundlich isotherm model is as follows:

$$log\, q_e = log\, k_F + \frac{1}{n} log\, C_e \quad \text{(Equation S6)}$$

Where, q_e represents equilibrium adsorption capacity (mg/g); k_F is adsorption equilibrium constant (mg/g) $(L/mg)^{1/n}$; n represents empirical constant; c_e is Solution Equilibrium Concentration (mg/L).

The Langmuir isotherm model is as follows:

$$\frac{c_e}{q_e} = \frac{1}{k_L} \frac{1}{q_{max}} + \frac{1}{q_{max}} c_e \quad \text{(Equation S7)}$$

Where, c_e is Solution Equilibrium Concentration (mg/L); q_e represents equilibrium adsorption capacity (mg/g); k_L is adsorption equilibrium constant (L/mg); q_{max} represents maximum adsorption capacity (mg/g).

The relationship temperature and adsorption thermodynamic computation is as follows:

$$k_a = \frac{q_e}{c_e} \quad \text{(Equation S8)}$$

$$\Delta G = -RT\, ln\, k_a \quad \text{(Equation S9)}$$

$$ln\, k_a = \frac{\Delta H}{T} + \frac{\Delta S}{R} \quad \text{(Equation S10)}$$

Where k_a is the adsorption distribution coefficient; c_e is Solution Equilibrium Concentration (mg/L); qe represents equilibrium adsorption capacity (mg/g); T refers to the temperature (K); R is the universal gas constant (R =8.314 J/mol/K).

The specific capacitance in the experiment can be calculated by the following formula:

$$C_s = \frac{I\Delta t}{m\Delta E} \quad \text{(Equation S11)}$$

Where, C_s represents the specific capacitance of the electrode (F/g); I represents the discharge current (A); Δt represents the discharge time (s); ΔE represents the potential drop during the charging and discharging process (V); m represents the mass of the electroactive material on the electrode (g)

The voltammetric response of an electrode at various sweep rates can be generally expressed as follows:

$$i = av^b \quad \text{(Equation S12)}$$

Where, *i* represents both anodic and cathodic peak currents (A); ν is scan rate (mV/s); a and b are arbitrary coefficients.

Diffusion and capacitive contribution to the current response can be analyzed as follow:

$$i(v) = k_1 v + k_2 v^{\frac{1}{2}} \quad \text{(Equation S13)}$$

where, *i* represents the current density (A/g); ν is scan rate (mV/s); k_1 and k_2 are adjustable parameters.

1.3 Molecular simulation computational details

All simulations were performed in the GROMACS[1] (version 2020.6) simulation package. The parameters for γ-CD-MOF used the Universal force field (UFF)[2], which covers the whole periodic table. The TIP3P water model was used to describe the water molecules, and the Methyl Green molecules used the General Amber force field (GAFF). The same numbers of 20 MG molecules were randomly inserted into the systems with γ-CD-MOF before solvation of around 6800 Methanol molecules. After thousands of steps of energy minimization, molecular dynamics simulations under the iso-thermo and iso-baric (NPT) ensemble were performed to equilibrate the system under 1 atm along the norm of the surface at 298.15 K. Subsequent 100 ns production runs were carried out under the carnonic ensemble for each system using the nose-hoover coupling method. A cutoff scheme was implemented at 1.2 nm for the non-bonded interactions, and the Particle Mesh Ewald method with a fourierspacing of 0.1 nm was applied for the long-range electrostatic interactions. All covalent bonds with were constraint using the LINCS algorithm.

2. Supplementary experimental results

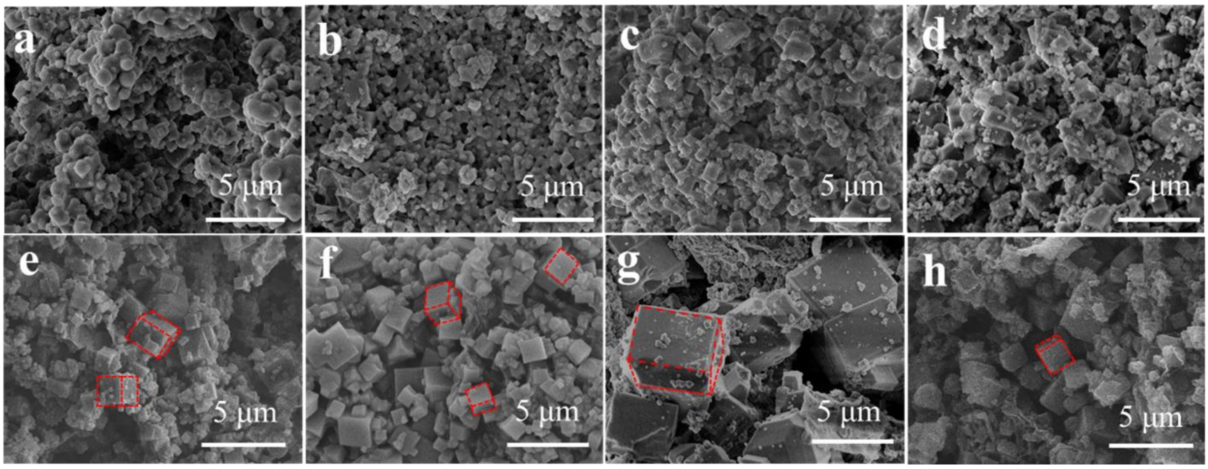

Figure S1. SEM images of γ-CD-MOF/GO prepared at different GO concentrations: a) 0, b) 0.1, c) 0.5, d) 1, e) 2, f) 3, g) 4 $g \cdot L^{-1}$. h) SEM image of γ-CD-MOF/GO/MG.

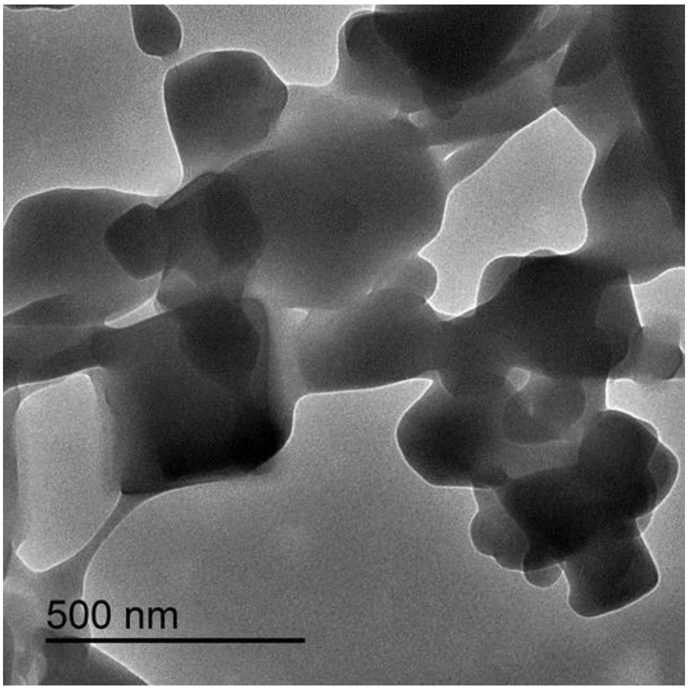

Figure S2. TEM image of γ-CD-MOF prepared without GO by the microwave procedure.

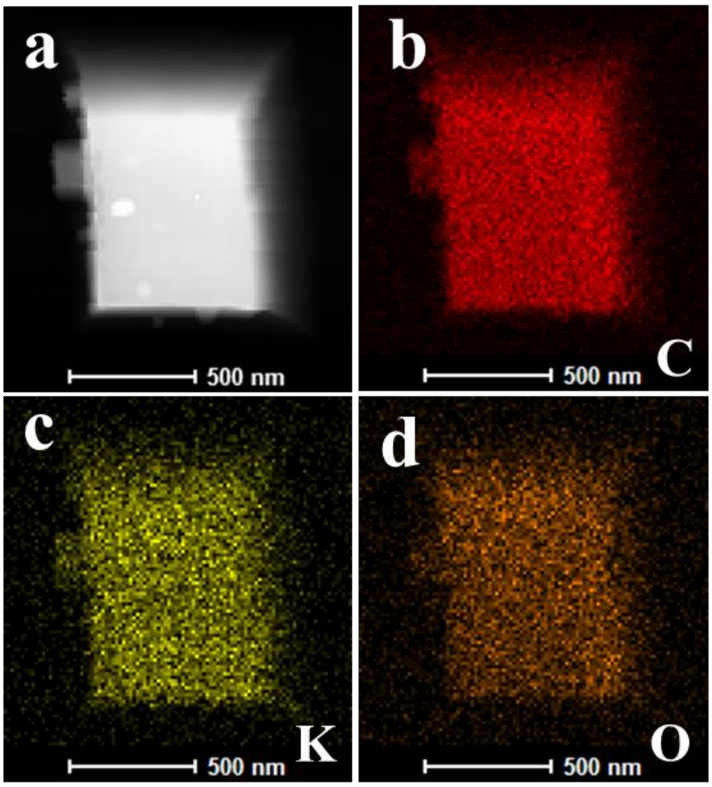

Figure S3. The elemental mapping images of γ-CD-MOF/GO.

Cubic crystal γ-CD-MOF was observed by TEM (Figure S3). The mapping of γ-CD-MOF/GO at this GO concentration of 3 g/L illustrated that the elements are evenly dispersed.

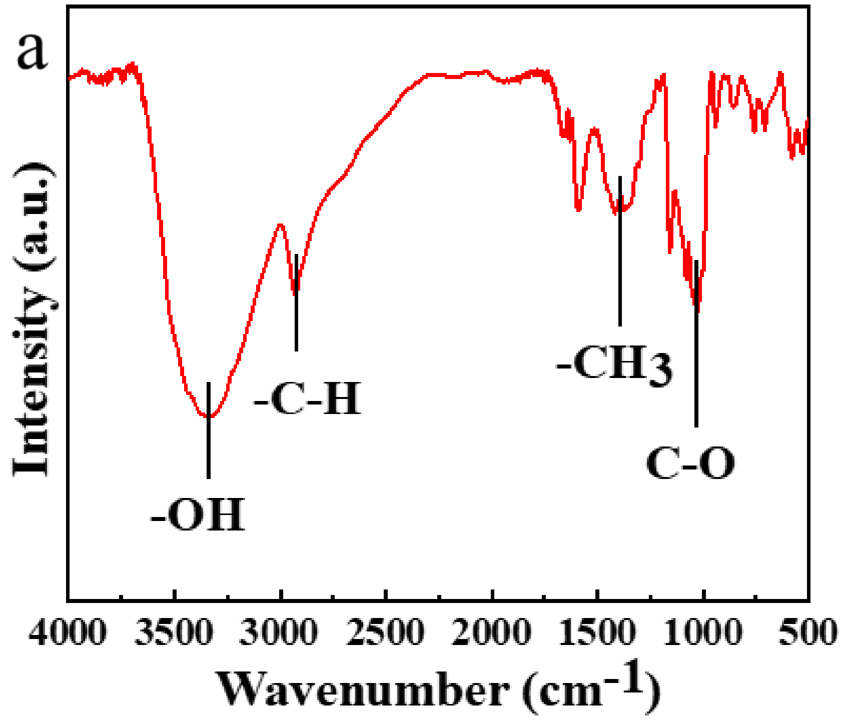

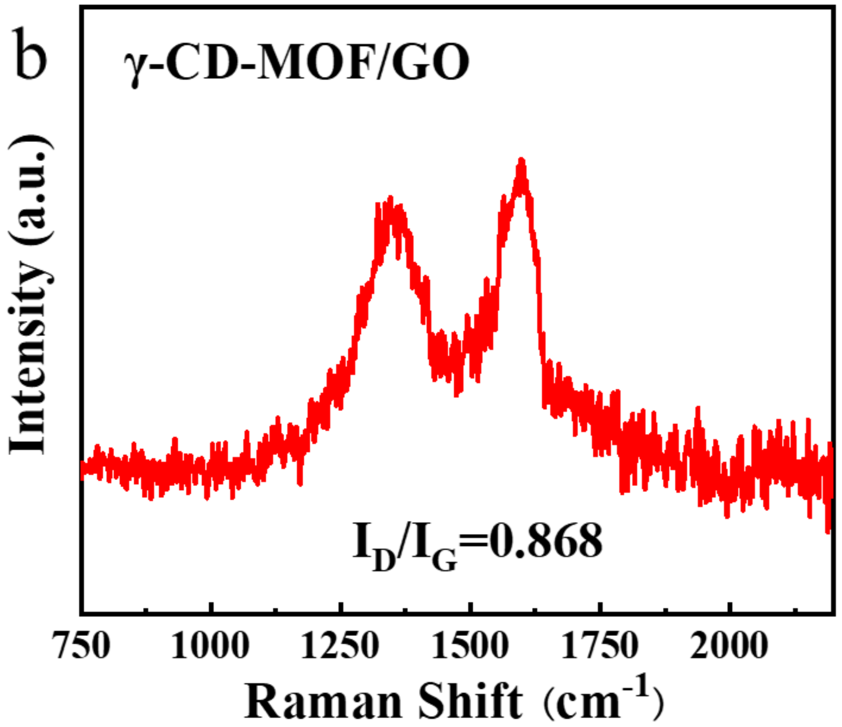

Figure S4. (a) FTIR spectrum and (b) Raman spectrum of γ-CD-MOF/GO.

The FTIR spectrum of γ-CD-MOF/GO showed the typical absorption peak of CD. From the Raman spectrum of γ-CD-MOF/GO (Figure S4 b), the relatively low value of I_D/I_G proved the partial reduction of GO.

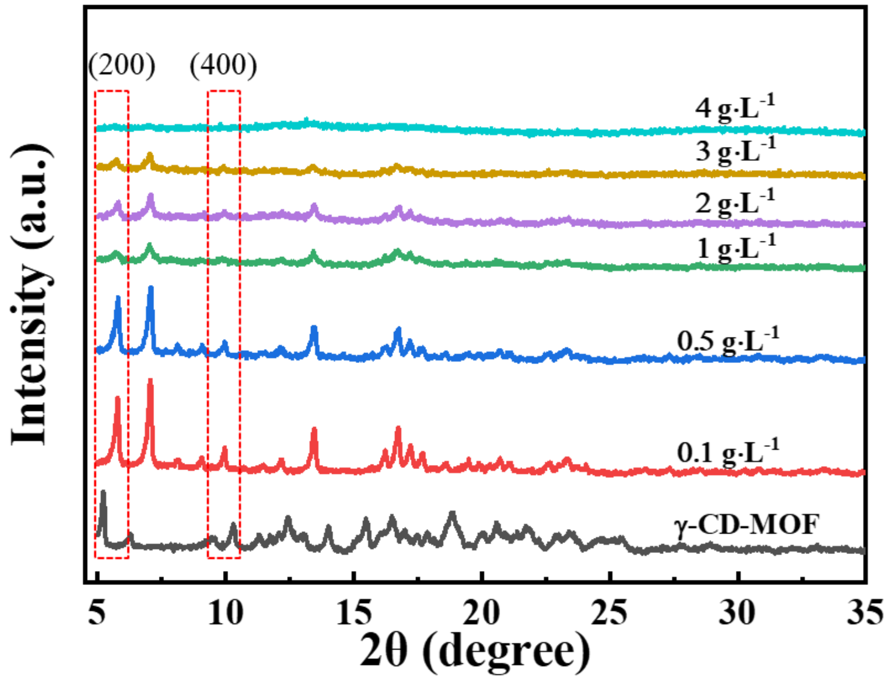

Figure S5. XRD patterns of γ-CD-MOF/GO and γ-CD-MOF.

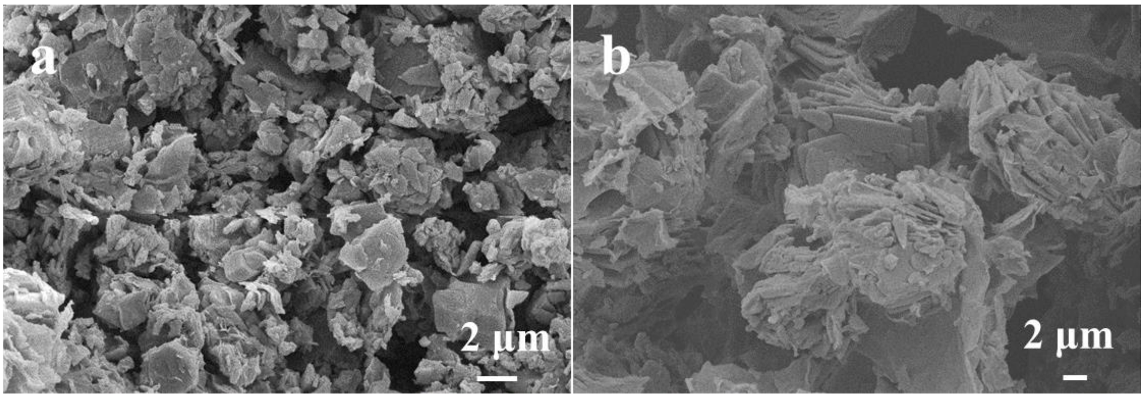

Figure S6. SEM images of (a) α-CD-MOF/GO and (b) β-CD-MOF/GO.

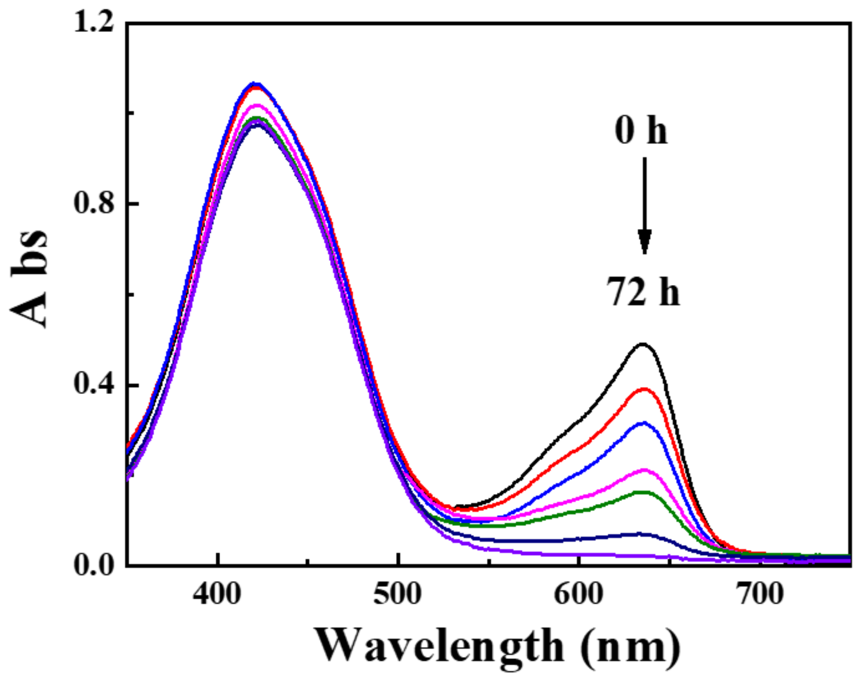

Figure S7. UV-Vis spectra of MO+MG adsorbed by γ-CD-MOF/GO in 72 h.

We mixed MO and MG to get 20 ml of mixed solution and used it to study the selective adsorption effect. Because the UV characteristic peaks of MB coincided with those of MG, no MB was added to prevent interaction. The UV absorbance test of MO and MG mixed solution at 72 hours was shown in the picture. It was seen that the change of the characteristic peak intensity of MO at 430 nm was relatively small, while the characteristic peak intensity of MG at 640 nm hardly changed. It was meant that MG was almost completely adsorbed. It can be concluded that γ-CD-MOF/GO has high selective adsorption effect on MG.

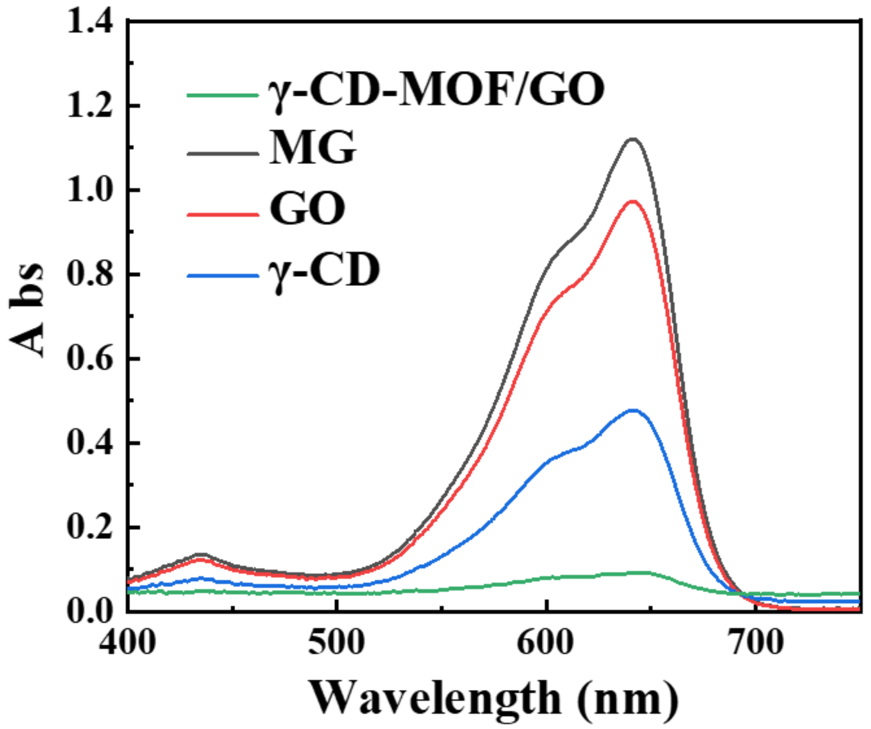

Figure S8. UV-Vis spectra of MG adsorbed by GO, γ-CD, and γ-CD-MOF/GO in 72 h.

The UV-Vis spectra of MG adsorbed by GO, γ-CD, and γ-CD-MOF/GO, respectively. It was seen that the characteristic peak intensity of MG was at 640 nm. The results indicated that GO did not have a good adsorption effect on MG.

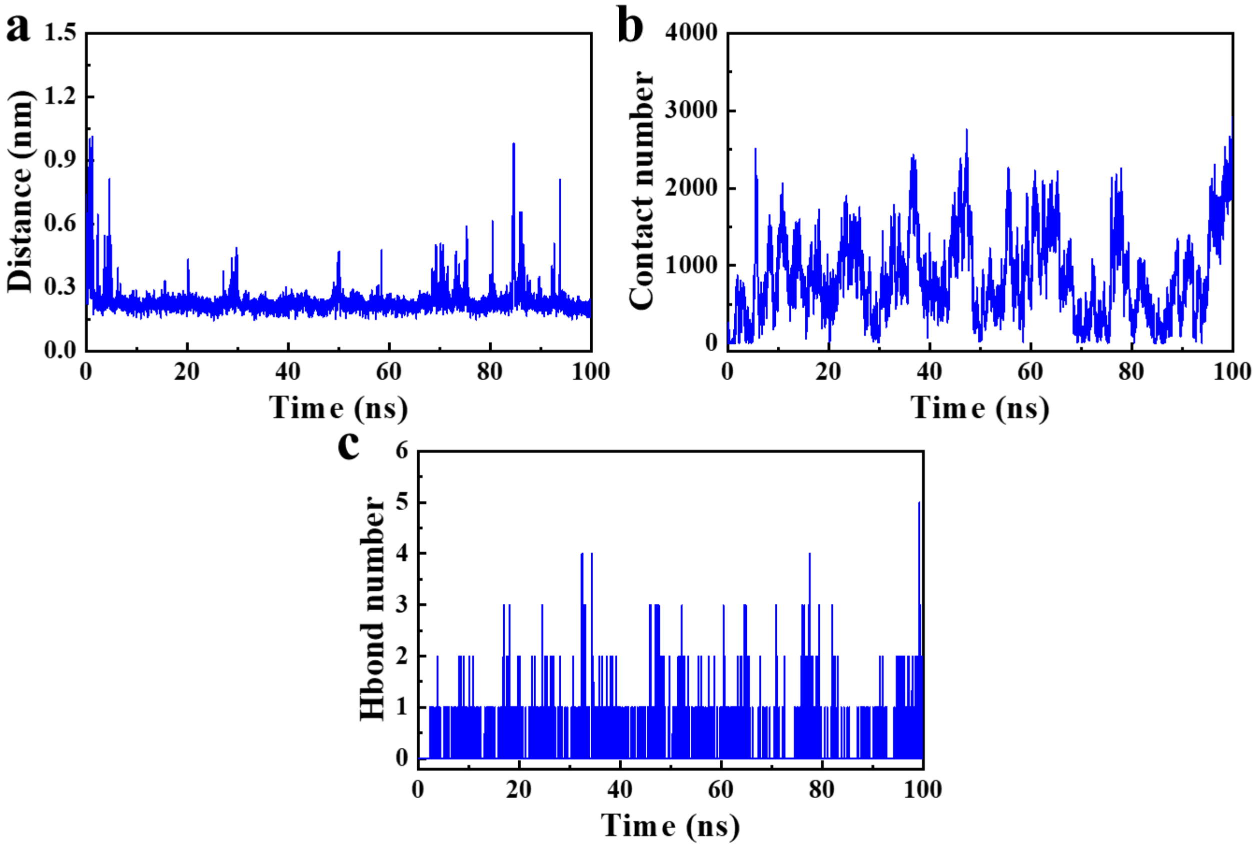

Figure S9. a) The distance, b) The number of atomic contacts, c) The number of hydrogen bonds between the γ-CD-MOF molecule and the methylene green molecule in the system.

The theoretical adsorption of MG on γ-CD-MOF was investigated by establishing a molecular model. In the simulation system, methanol was used as a solvent. The changes of intermolecular distance, number of intermolecular contact and hydrogen bond were observed in 100 ns. According to the results of the simulation, firstly, the distance between γ-CD-MOF molecules and MG in the system was calculated. From the changes in Figure S9a, it can be found that the distance between MG and cyclodextrin gradually decreased. Then, the number of atomic contacts between γ-CD-MOF molecules and MG in the system was calculated. It was found that the number of contacts between CD and MG molecules increased as the simulation progressed (Figure S9b), indicating that CD has a certain adsorption effect on MG. The number of hydrogen bonds between the two molecules was further calculated (Figure S9c), and the revealed decrease may be more related to the conjugated interaction between the CD rings, indicating that the adsorption is mainly directed at the phenothiazine ring in MG, and it can be concluded that the cavity of cyclodextrin adsorbs MG 's phenothiazine ring internally.

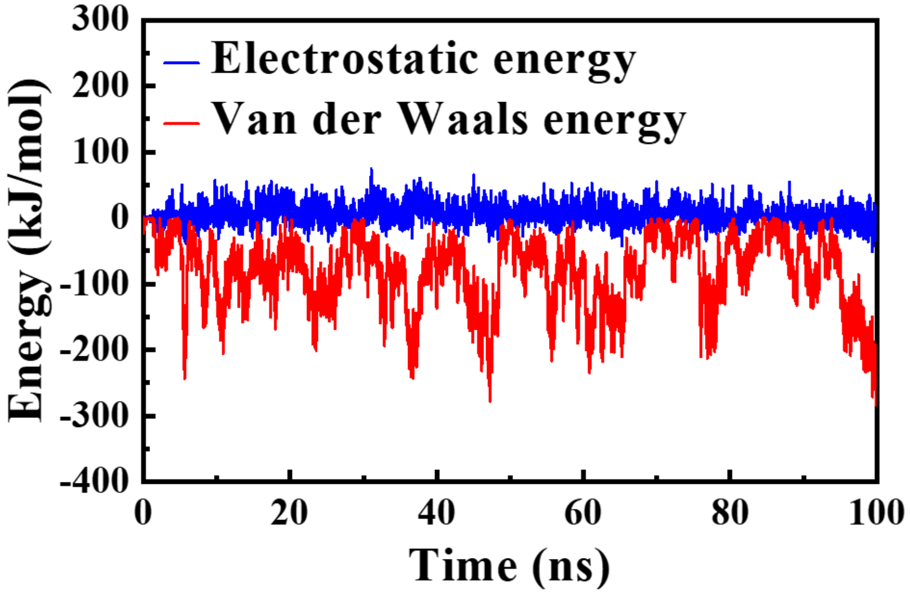

Figure S10. The electrostatic energy and van der Waals energy between the γ-CD-MOF molecule and the methylene green molecule in the system.

The interaction energy between CD and MG molecules was calculated (Figure S10). The electrostatic energy and van der Waals energy were calculated. The interaction between γ-CD-MOF and MG was mainly the stronger Van der Waals energy, as shown in the diagram. So Van der Waals energy was the main energy source of γ-CD-MOF adsorbing MG.

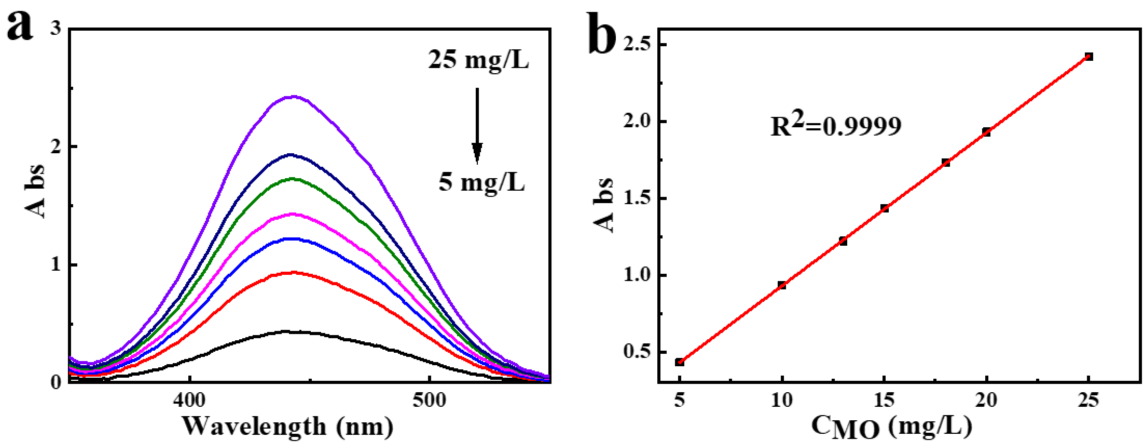

Figure S11. a) UV-Vis spectra of MO solution at different concentration (5-25 mg/L). b) Linear relationship graph between absorbance and C_{MO}.

In order to get the relationship between the concentration and the absorbency, we prepared the dye solution with different concentration and tested the UV absorbency. The absorbance of MO solution of 5 to 25 mg/L was shown in Figure S11a. Scatter plots of solution concentration and absorbance were obtained by peak values of different concentrations at the same wavelength. Then, solution concentrations and absorbance in Figure S11b were linearly fitted using Lambert-Beer law. The linear fitting constant R^2=0.9999 indicated a high degree of agreement between the actual test results and the theoretical data.

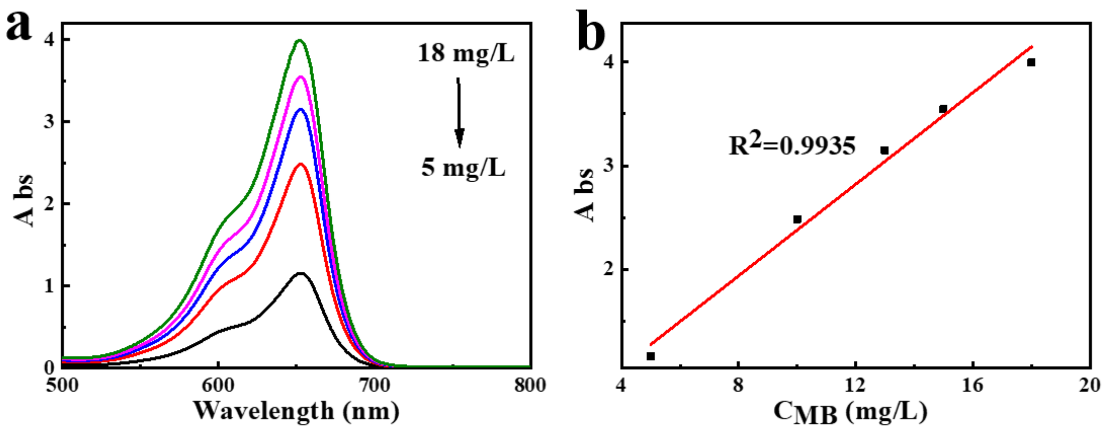

Figure S12. a) UV-Vis spectra of MB solution at different concentration (5-18 mg/L). b) Linear relationship graph between absorbance and C_{MB}.

Similarly, the relationship between the concentration of MB solution and UV absorbance was obtained using the methods described above. The absorbance of MB solution of 5 to 18 mg/L was shown in Figure S12a. Scatter plots of solution concentration and absorbance were obtained by peak values of different concentrations at the same wavelength. Then, solution concentrations and absorbance in Figure S12b were linearly fitted using Lambert-Beer law. The linear fitting constant R^2=0.9935 indicated a high degree of agreement between the actual test results and the theoretical data.

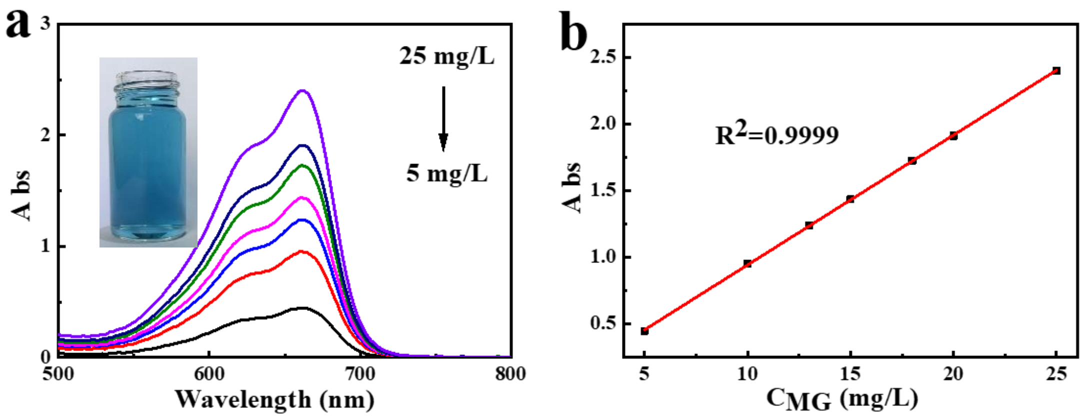

Figure S13. a) UV-Vis spectra of MG solution at different concentration (5-25 mg/L). b) Linear relationship graph between absorbance and C_{MG}.

The relationship between the concentration of MG solution and UV absorbance was obtained using the methods described above. The absorbance of MG solution of 5 to 25 mg/L was shown in Figure S13a. Scatter plots of solution concentration and absorbance were obtained by peak values of different concentrations at the same wavelength. Then, solution concentrations and absorbance in Figure b were linearly fitted using Lambert-Beer law. The linear fitting constant R^2=0.9999 indicated a high degree of agreement between the actual test results and the theoretical data (Figure S13b).

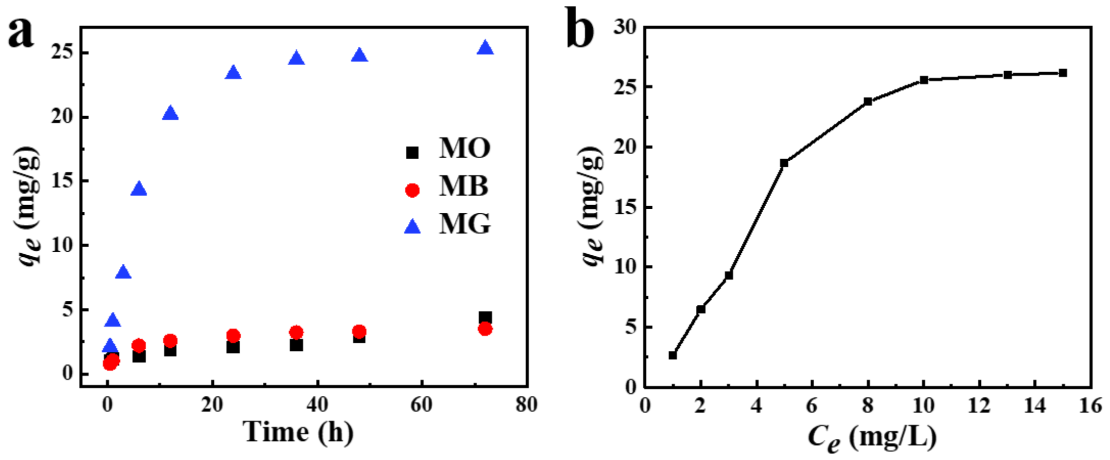

Figure S14. a) Adsorption capacity of γ-CD-MOF/GO to MO, MB, MG. b) Diagram of C_e and q_e.

In order to compare the adsorption effect of γ-CD-MOF/GO on three dyes more intuitively, the relationship between adsorption amount and time at different time as shown in Figure S14a. It was clearly seen that the amount of dye adsorbed by γ-CD-MOF/GO increased with time. Among them, the adsorption capacity of MG is the best.

Here, it was apparent that the MG adsorption property of γ-CD-MOF/GO increased more rapidly than MO and MB during the first 10 h, and the adsorption equilibrium was achieved after 20 h. At 72 hours, the amount of adsorption was stable and the maximum amount of adsorption was achieved. The adsorption capacity of γ-CD-MOF/GO to MO, MB and MG was 4.40 mg/g, 3.53 mg/g and 25.28 mg/g, respectively. The above results indicated that γ-CD-MOF/GO had selective adsorption for MG.

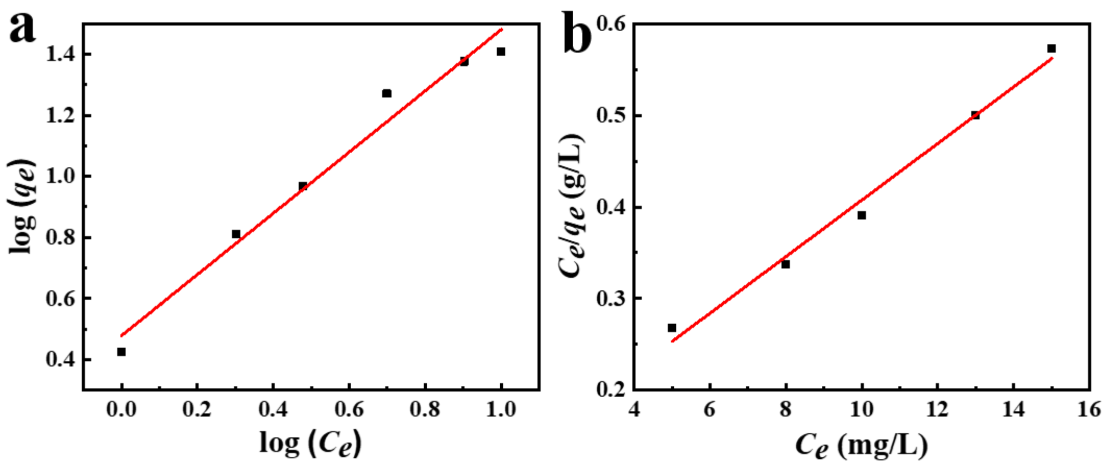

Figure S15. a) Freundlich isotherm model linear relationship, b) Langmuir isotherm model linear relationship for adsorption to MG.

The Freundlich and Langmuir adsorption isotherm models were used to study the isotherms of the adsorption process to MG. Figure S15a was Freundlich adsorption isotherm models as shown in (Supplementary Equation 6) and Figure S15b was Langmuir adsorption isotherm models as shown in (Supplementary Equation 7). They represented different types of adsorption[3]. The saturated adsorption capacity of γ-CD-MOF/Go for MG at different concentrations was calculated at room temperature. According to Equation 6 and Equation 7, the detailed data for the two isotherm models were provided in Supplementary Table 2. From the data in the table, the linear fitting constant R^2 for Freundlich adsorption isotherm models is relatively large at 0.9896. So, the adsorption process was more consistent with Freundlich adsorption isotherm models. The adsorption of γ-CD-MOF/GO to MG was a spontaneous process.

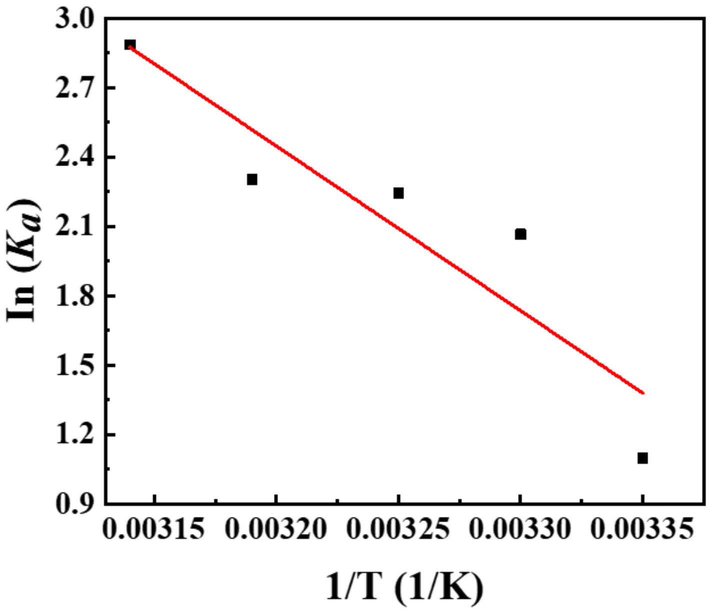

Figure S16. Thermodynamic relationship of In (K_a) versus 1/T.

In order to study the adsorption thermodynamics of γ-CD-MOF/GO to MG, 10 mg/L MG was adsorbed at different temperatures for 72 hours. We chosed 303 K, 308 K, 313 K, 318 K as the study temperature. Using the Equation 10, the detailed data was listed in Supplementary Table 3. It was shown that the Gibbs free energy (ΔG) was less than 0 and the adsorption process was a spontaneous process. This conclusion was consistent with the results of Freundlich adsorption isotherm models. The enthalpy changes (ΔH) and entropy change (ΔS) were also obtained, and the adsorption process was exothermic.[4] The γ-CD-MOF/GO adsorbed MG in pore size through electrostatic, Van der Waals force and supramolecular interactions between MG and hydroxyl and carboxyl groups at the adsorption sites.

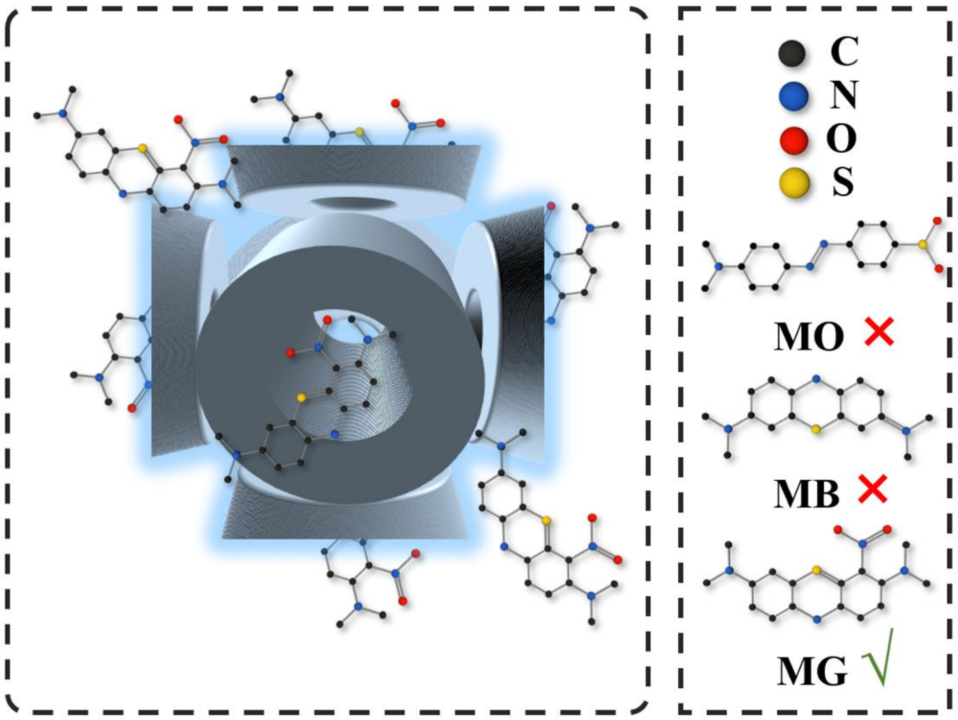

Figure S17. The effective sites for the adsorption of γ-CD-MOF/GO to MG.

As shown in Figure S17, γ-CD-MOF/GO could effectively adsorb MG with its own hydrophobic cavity. According to the experimental data and molecular simulation results, the adsorption of MG on γ-CD-MOF was mainly through van der Waals force and electrostatic interaction. The phenothiazine ring of MG could be effectively adsorbed by γ-CD-MOF, and the MG molecules were clustered around the γ-CD-MOF. MG and γ-CD-MOF/GO showed strong supramolecular interactions.

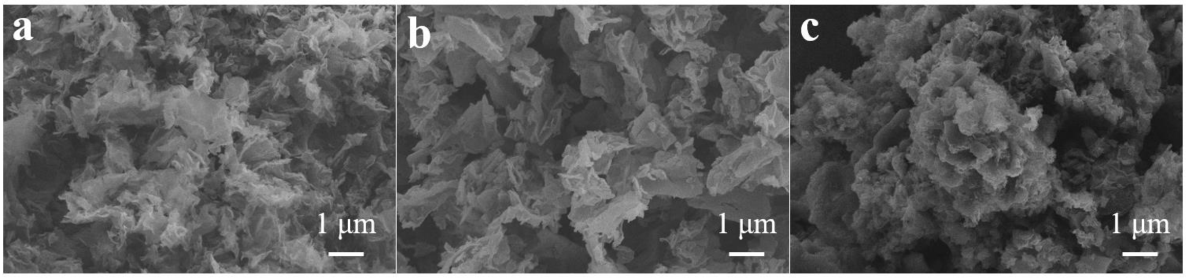

Figure S18. SEM images of a) α-CD-MOF/GO-600, b) β-CD-MOF/GO-600, c) γ-CD-MOF/GO-600.

Figure S18 showed the SEM images of α-, β-, and γ-CD-MOF/GO-derived porous carbons, respectively. The results showed the layered porous carbons derived from α-CD-MOF/GO, β-CD-MOF/GO, and γ-CD-MOF/GO. The different kinds of CDs had no obvious effect on the derived porous carbon structure.

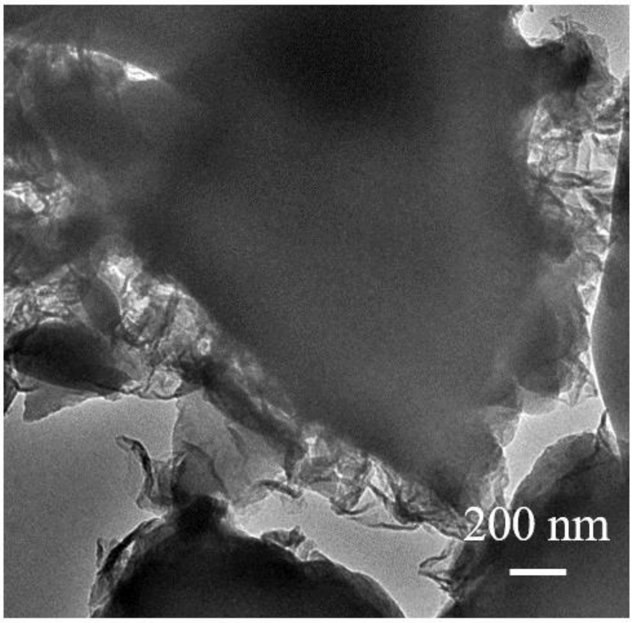

Figure S19. TEM image of γ-CD-MOF/GO-600.

The structure of porous carbon γ-CD-MOF/GO-600 was observed by TEM. The result was consisting with the SEM image. The calcined GO were distributed around the porous carbon, and the folded surface greatly increased the specific surface area[5].

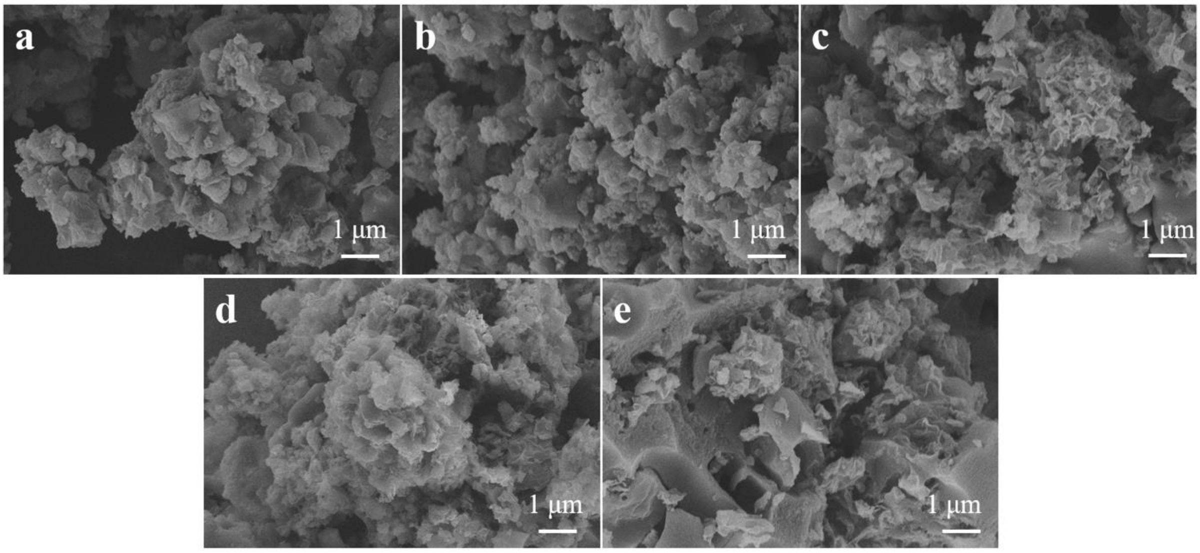

Figure S20. SEM images of a) γ-CD-MOF/GO-300, b) γ-CD-MOF/GO-400, c) γ-CD-MOF/GO-500, d) γ-CD-MOF/GO-600, e) γ-CD-MOF/GO-700.

The γ-CD-MOF/GO prepared by 3 g/L GO was calcined at different temperatures (300-700°C). From Figure S20a and b, the porous carbon γ-CD-MOF/GO-300 and γ-CD-MOF/GO-400 were obviously not sufficiently carbonized, and the skeleton of MOF was not completely damaged, and γ-CD-MOF/GO-300 and γ-CD-MOF/GO-400 retained partly the precursor morphology. The graphene distribution on the surface of the calcined product was the most uniform at 600°C. As the temperature increased, as shown in Figure S20c-e, the structure of γ-CD-MOF/GO was destroyed, and the surface of γ-CD-MOF/GO was severely corroded due to the evaporation of metal ions and the decomposition of organic matter during the high temperature process, forming porous structure. The structure might be beneficial to realize the rapid transfer of ions in the electrochemical process and improve the electrochemical performance[6].

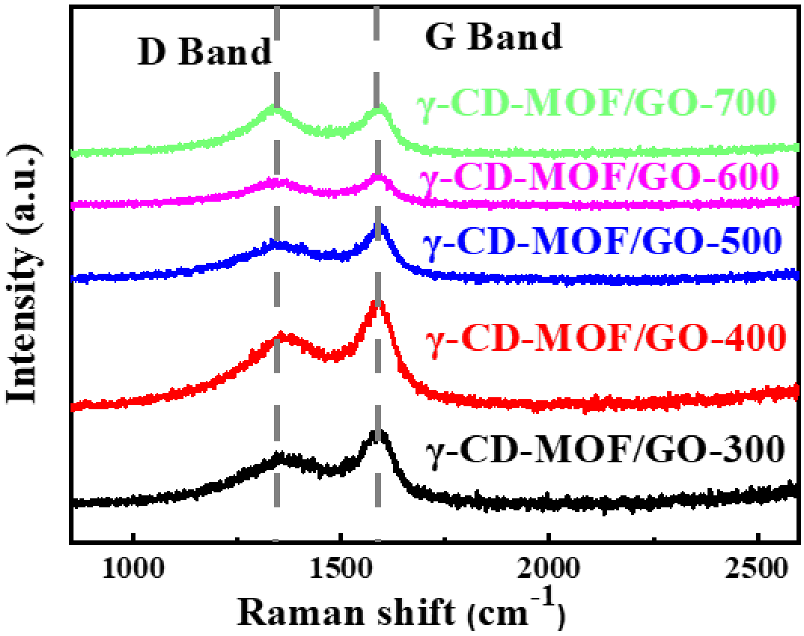

Figure S21. Raman spectra of γ-CD-MOF/GO prepared with different temperature.

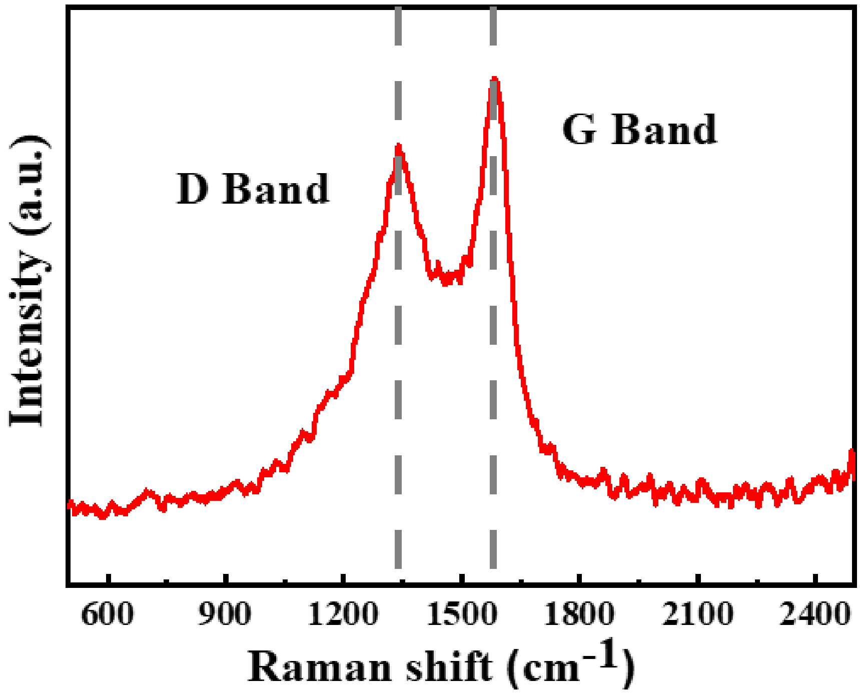

Figure S22. Raman spectra of γ-CD-MOF/GO/MG-600.

To investigate the effect of adsorption on the surface disorder of calcined products, γ-CD-MOF/GO/MG-600 was tested in Raman spectra. Two distinct characteristic peaks, D-band and G-band represented the disordered carbon structure and graphite carbon structure, respectively[7]. The degree of surface defect was obtained by the strength or area ratio of D-band and G-band. By calculation, the I_D/I_G of γ-CD-MOF/GO/MG-600 was similar to the result of γ-CD-MOF/GO-600. It was concluded that adsorption had little effect on the degree of surface defects.

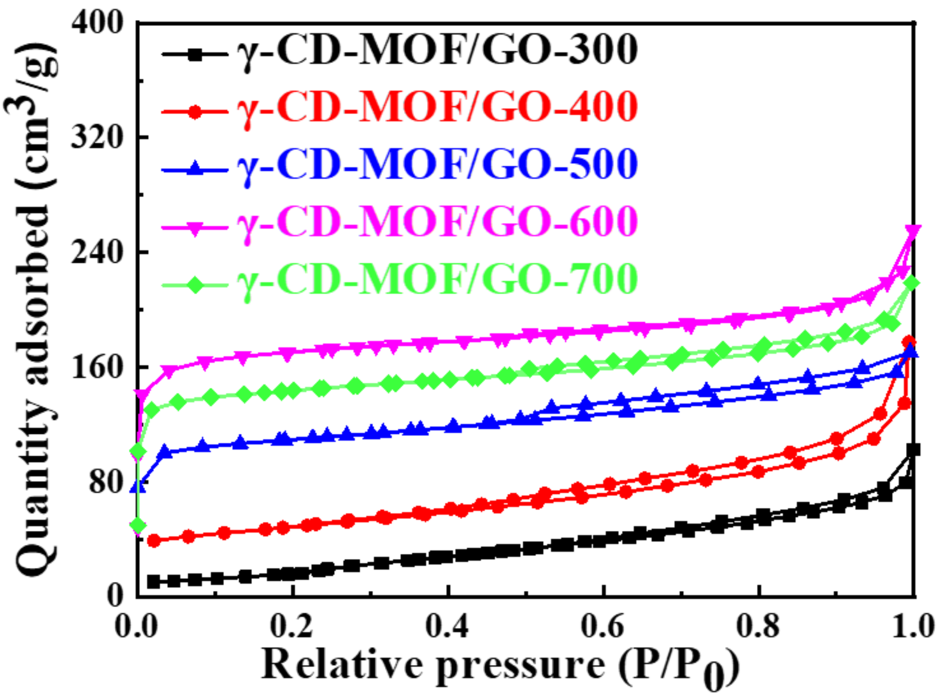

Figure S23. N_2 absorption–desorption isotherms of γ-CD-MOF/GO prepared with different temperature.

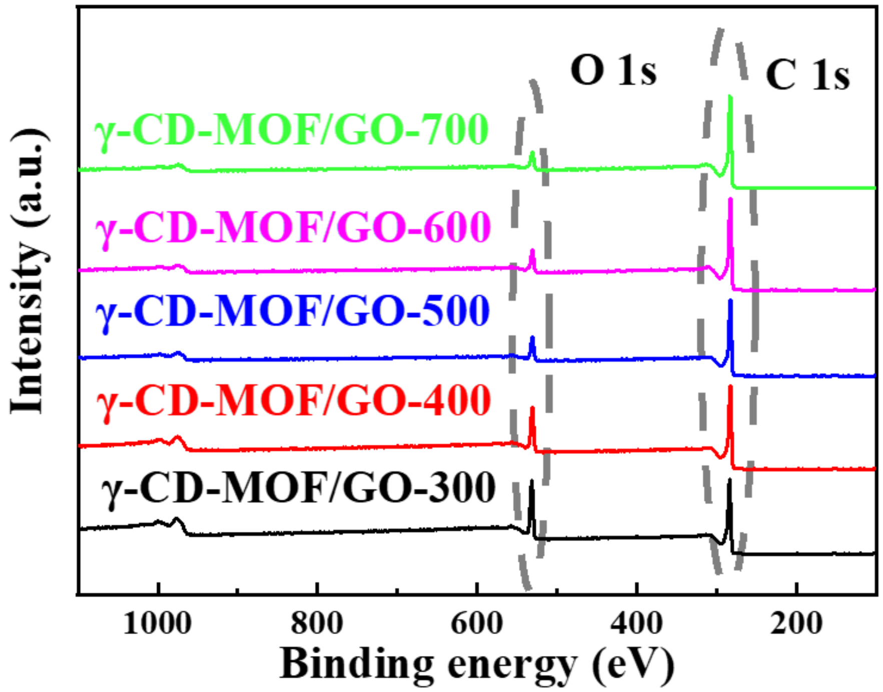

Figure S24. XPS spectra of γ-CD-MOF/GO prepared with different temperature.

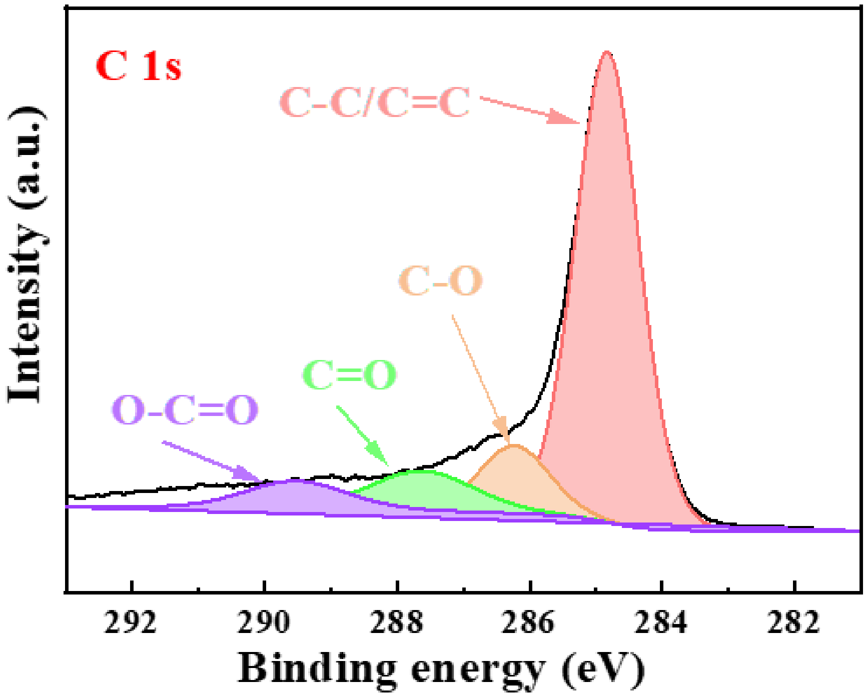

Figure S25. C 1s XPS spectrum of γ-CD-MOF/GO-600.

The contents and functional groups of elements were studied by XPS. As shown in the XPS spectrum of γ-CD-MOF/GO-600, the main four peaks of C elements were C-C/C=C at 284.8 eV, C-O at 286 eV, C=O at 288.5 eV, and O-C=O at 289 eV, respectively. In addition to the carbon-carbon bonds, there were a large number of carbon-oxygen bonds, mainly derived from the rich oxygen-containing functional groups on the surface of γ-CD and GO. A large number of oxygen-containing functional groups in the adsorption process provided a large number of active sites.

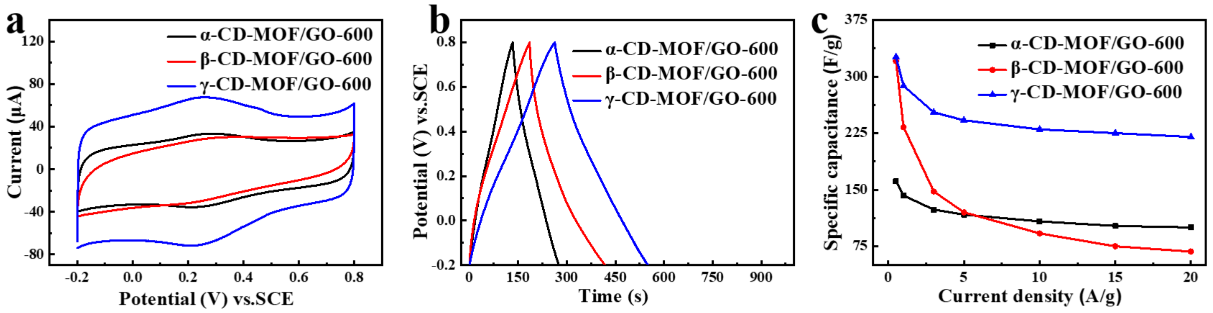

Figure S26. a) CV curves (scan rates: 50 mV/s), b) GCD curves (current densities: 1 A/g), c) Specific capacitance at different current densities (0.5-20 A/g) of the electrodes modified by α-CD-MOF/GO-600, β-CD-MOF/GO-600, γ-CD-MOF/GO-600 in 1 mol/L H_2SO_4.

The effects of α, β, γ-CD on the electrochemical properties of the derived porous carbon were compared by electrochemical measurements in a three-electrode system. The α-CD-MOF/GO, β-CD-MOF/GO, γ-CD-MOF/GO were calcined at 600°C to form porous carbon α-CD-MOF/GO -600, β-CD-MOF/GO -600, γ-CD-MOF/GO-600. Figure S26a displayed the CV curves at 50 mV·s^{-1}, and γ-CD-MOF/GO-600 had the largest area indicating its larger specific capacitance[8]. Figure S26b showed the GCD curves at 1A·g^{-1}. The γ-CD-MOF/GO-600 has the longest charge-discharge time and the best electrochemical performance. Comparing the specific capacitance of three kinds of porous carbon materials under different current density in Figure S26c, γ-CD-MOF/GO-600 had the highest specific capacitance. At the current density of 0.5 A·g^{-1}, the specific capacitances of α, β, γ-CD-MOF/GO-600 were 261.2 F·g^{-1}, 320.5 F·g^{-1}, and 326 F·g^{-1}, respectively. The capacitance retention rate of γ-CD-MOF/GO-600 was 67.5%.

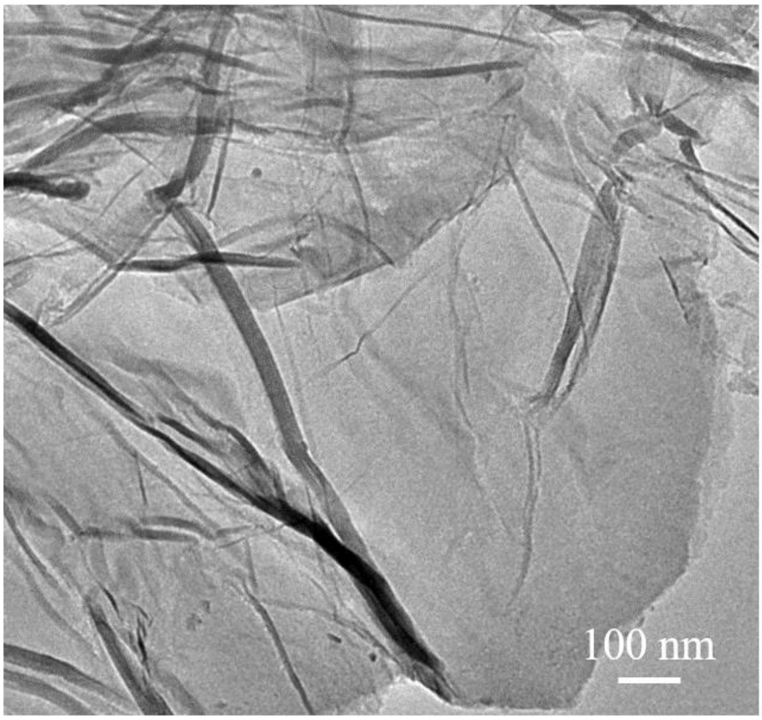

Figure S27. TEM image of GO-600.

To further verify that the distribution around the above porous carbon is the calcined product of graphene. We calcined GO at 600°C to obtain the structure shown in the diagram. GO is calcined to maintain a two-dimensional sheet structure**.**

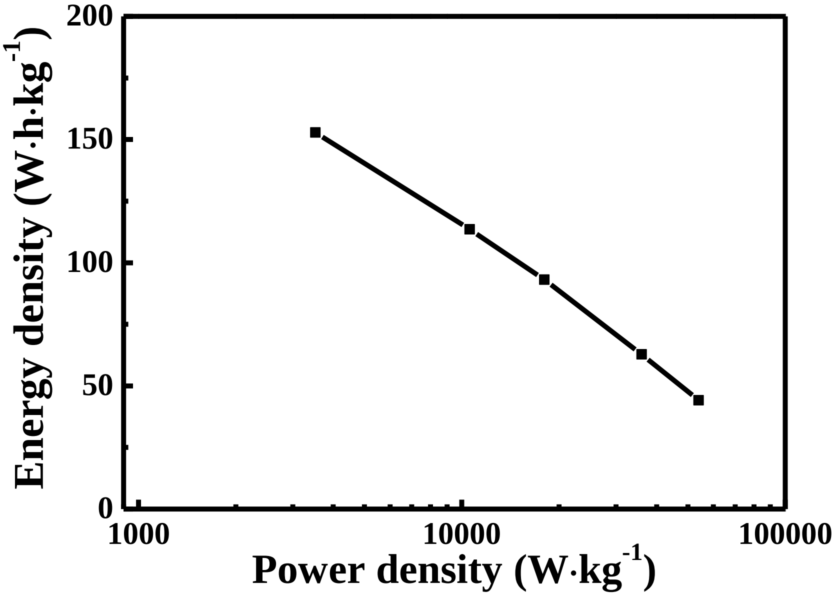

Figure S28. The corresponding Ragone plots related to specific energy and specific power of the γ-CD-MOF/GO/MG-600 SC.

Table S1. Parameters calculated from adsorption kinetics model fitting.

Dye	Experimental q_{exp} (mg/g)	Pseudo-first-order kinetics			Pseudo-second- order kinetics			Intraparticle diffusion model	
		q_e (mg/g)	k_f (/h)	R^2	q_e (mg/g)	K_s (g/mg/h)	R^2	K_w (mg/g/h$^{1/2}$)	R^2
MO	4.40	3.30	0.015	0.9464	4.10	0.020	0.8255	0.369	0.8834
MB	3.53	2.19	0.053	0.9568	3.62	0.077	0.9976	0.348	0.8872
MG	25.28	19.18	0.081	0.9632	27.78	0.006	0.9989	3.125	0.8418

Three adsorption kinetic models were established, which are quasi-first-order, quasi-second-order and intra-particle diffusion kinetic models. The three kinetic models represented different adsorption processes. The adsorption process accorded with the quasi-first-order kinetic model, which showed that the adsorption process was a pure electrostatic adsorption. The adsorption process followed a quasi-second-order kinetic model, which suggested that there are more complex intermolecular force besides electrostatic interactions. The adsorption process accords with the diffusion model in the particle, which showed that there were two steps in the main atmosphere of adsorption. One was the adsorption on the surface, and the other was the slow diffusion in the pore. After calculation, the detailed kinetic model data were shown in the table. The adsorption of MO on γ-CD-MOF/GO was shown to be a simple electrostatic process by observing the linear fitting and constant R^2, which was more in accordance with the quasi-first-order kinetic model. However, due to the involvement of oxygen-containing functional groups, the adsorption of MO was relatively low. The results of MB, MG adsorption agreed with the quasi-second-order kinetic model, indicating that the supramolecular interaction of the two dyes by the cavity of CD leaded to more intermolecular force. The saturated adsorption capacity obtained from the experiment was almost the same as the theoretical adsorption capacity, which proved the reliability of our experimental results.

Table S2. Parameters calculated from adsorption isotherm model fitting to MG.

Model	Parameters	
**Freundlich**	K_F (mg/g) (L/mg)$^{1/n}$	3.013
	R^2	0.9658
**Langmuir**	q_{max} (mg/g)	28.32
	K_L (L/mg)	0.3132
	R^2	0.9896

Table S3. Thermodynamic parameters of adsorption.

T	In Ka	ΔG (kJ/mol)	ΔH (kJ/mol)	ΔS (kJ/mol K)
298 K	1.099	-2.723		
303 K	2.066	-5.204		
308 K	2.245	-5.749	59.162	0.212
313 K	2.304	-5.996		
318 K	2.886	-7.630		

Table S4. BET surface areas and average pore size of γ-CD-MOF/GO-n prepared at different temperature: 300, 400, 500, 600 and 700 °C.

Temperature (°C)	Surface area (m^2/g)	Average pore size (nm)
300	54.70	6.8
400	170.09	5.7
500	382.04	2.9
**600**	**594.61**	**2.7**
700	501.14	2.9

Table S5. C and O elemental contents of γ-CD-MOF/GO-n prepared at different temperature: 300, 400, 500, 600 and 700 °C obtained from XPS spectra.

Temperature (°C)	C Percentage（%）	O Percentage（%）
300	76.72	23.28
400	82.00	18.00
500	88.24	11.76
600	90.70	9.30
700	91.35	8.48

Using the XPS test results, the element types in the material can be obtained. The table shows the content of C and O in 3 g/L GO-derived porous carbon γ-CD-MOF/GO-n at different calcination temperatures. According to their XPS spectra, the derived porous carbon mainly contained C and O elements. With the increase of calcination temperature, the content of C increased obviously and reached 91.35% at 700°C. In addition to element C, there was also a large amount of element O in the calcined product due to the presence of GO. With the increase of temperature, the oxygen-containing functional groups were destroyed, and the content of O decreased. The presence of O element provided active sites and improved the electrochemical properties of the materials, therefore, the suitable content of O element in γ-CD-MOF/GO-600 became the basis of better electrochemical performance, and the high content of C element increased the conductivity of the material.

Table S6. Specific capacitance of various reported carbon-based cathode materials.

**Cathode**	**Voltage window (V)**	**Specific capacitance (F/g)**	**Electrolyte**	**Current densities (A/g)**	**Ref.**
γ-CD-MOF/GO/MG-600	-0.2~0.8	501	1 M H_2SO_4	0.5	This work
MOG-La-Fe-700	-1.0~0	241.3	1 M Na_2SO_4	1	Ref [9]
NOC-800	-1.0~0	391	6 M KOH	1	Ref [10]
KNPG	-1.0~0	201	6 M KOH	0.5	Ref [11]
NOHPC	-0.1~0.9	328.3	1 M H_2SO_4	0.625	Ref [12]
DPC-T	-1.0~0	320.7	6 M KOH	1	Ref [13]
N-GQD/3DrGO	-1.0~1.0	361	1 M Na_2SO_4	2	Ref [14]
NPC-3-850	-0.2~0.8	334	1 M H_2SO_4	1	Ref [15]

3. Supplementary References

[1] B. Hess, C. Kutzner, D. van der Spoel, E. Lindahl, *J. Chem. Theory Comp.* **2008**, *4* (3), 435.
[2] A. K. Rappe, C. J. Casewit, K. S. Colwell, W. A. G. III, W. M. Skiff, *J. Am. Chem. Soc.* **1992**, *114* (25), 10024.
[3] W. Tang, H. Zhang, X. Yang, Z. Dai, Y. Sun, H. Liu, Z. Hu, X. Zheng, *Appl. Catal. B* **2023**, *320*, 121952.
[4] Z. Cui, J. Wu, Y. Xu, T. Wu, H. Li, J. Li, L. Kang, Y. Cai, J. Li, D. Tian, *Chem. Eng. J.* **2023**, *451*, 138371.
[5] C. Xu, W. Yang, G. Ma, S. Che, Y. Li, Y. Jia, N. Chen, G. Huang, Y. Li, *Small* **2022**, 2204375.
[6] H. Wu, W. Yuan, X. Yuan, L. Cheng, *Energy Storage Mater.* **2022**, *50*, 514.
[7] H. Zhou, Z. Ren, C. Xu, L. Xu, C. Lee, *Nano Micro Lett.* **2022**, *14* (1), 207.
[8] M. Zhong, M. Zhang, X. Li, *Carbon Energy* **2022**, *4* (5), 950.
[9] Y. Zhang, J. Ding, W. Xu, M. Wang, R. Shao, Y. Sun, B. Lin, *Chem. Eng. J.* **2020**, *386*, 124030.
[10] M. Wang, B. Liu, H. Chen, D. Yang, H. Li, *ACS Sustain. Chem. Eng.* **2019**, *7* (13), 11219.
[11] L. Zheng, K. Xia, B. Han, C. Zhou, Q. Gao, H. Wang, S. Pu, J. Wu, *ACS Appl. Nano Mater.* **2018**, *1* (12), 6742.
[12] W. Teng, Q. Zhou, X. Wang, H. Che, Y. Du, P. Hu, H. Li, J. Wang, *Appl. Surface Sci.* **2021**, *566*, 150613.
[13] D. Geng, S. Zhang, Y. Jiang, Z. Jiang, M. Shi, J. Chang, S. Liang, M. Zhang, J. Feng, T. Wei, Z. Fan, *J. Mater. Chem. A* **2022**, *10* (4), 2027.
[14] T. A. N. Bui, T. G. Nguyen, W. Darmanto, R.-A. Doong, *Electrochimi. Acta* **2020**, *361*, 137018.
[15] M. Qian, Z. Wang, Z. Li, J. Xu, P. Sun, J. Lin, T. Lin, F. Huang, *Microporous Mesoporous Mater.* **2019**, *286*, 18.
